# The Phytochemistry and Pharmacology of *Tulbaghia*, *Allium*, *Crinum* and *Cyrtanthus*: ‘Talented’ Taxa from the Amaryllidaceae

**DOI:** 10.3390/molecules27144475

**Published:** 2022-07-13

**Authors:** Cynthia Amaning Danquah, Prince Amankwah Baffour Minkah, Theresa A. Agana, Phanankosi Moyo, Michael Ofori, Peace Doe, Sibusiso Rali, Isaiah Osei Duah Junior, Kofi Bonsu Amankwah, Samuel Owusu Somuah, Isaac Newton Nugbemado, Vinesh J. Maharaj, Sanjib Bhakta, Simon Gibbons

**Affiliations:** 1 Department of Pharmacology, Faculty of Pharmacy and Pharmaceutical Sciences, College of Health Sciences, Kwame Nkrumah University of Science and Technology, PMB, Kumasi, Ghana; p.minkah@kccr.de (P.A.B.M.); michof2825@gmail.com (M.O.); nugbemadokorbla@gmail.com (I.N.N.); 2 Global Health and Infectious Disease Research Group, Kumasi Centre for Collaborative Research in Tropical Medicine, College of Health Sciences, Kwame Nkrumah University of Science and Technology, PMB, Kumasi, Ghana; 3 Department of Pharmaceutics, Faculty of Pharmacy and Pharmaceutical Sciences, College of Health Sciences, Kwame Nkrumah University of Science and Technology, PMB, Kumasi, Ghana; tessyagana4christ@gmail.com; 4 Department of Chemistry, University of Pretoria, Pretoria 0028, South Africa; u13386842@tuks.co.za (P.M.); u21749745@tuks.co.za (S.R.); vinesh.maharaj@up.ac.za (V.J.M.); 5 Department of Pharmaceutical Sciences, Dr. Hilla Limann Technical University, Wa P.O. Box 553, Ghana; 6 Department of Pharmaceutical Sciences, School of Pharmacy, Central University, Accra, Ghana; pdoe@central.edu.gh; 7 Department of Optometry and Visual Science, College of Science, Kwame Nkrumah University of Science and Technology, PMB, Kumasi, Ghana; oseiduahisaiah@gmail.com; 8 Department of Biomedical Sciences, University of Cape Coast, Cape Coast, Ghana; kamankwah@stu.ucc.edu.gh; 9 Department of Pharmacy Practice, School of Pharmacy, University of Health and Allied Sciences, Ho, Ghana; sosomuah@uhas.edu.gh; 10 Department of Biological Sciences, Institute of Structural and Molecular Biology, Birkbeck, University of London, Malet Street, London WC1E 7HX, UK; s.bhakta@bbk.ac.uk; 11 The Centre for Natural Products Discovery (CNPD), Liverpool John Moores University, Liverpool L3 3AF, UK; s.gibbons@ljmu.ac.uk

**Keywords:** Amaryllidaceae, alkaloids, *Allium*, *Crinum*, *Tulbaghia*, *Cyrtanthus*, phytochemicals, natural products, pharmacological activity, drug discovery

## Abstract

Amaryllidaceae is a significant source of bioactive phytochemicals with a strong propensity to develop new drugs. The genera *Allium*, *Tulbaghia*, *Cyrtanthus* and *Crinum* biosynthesize novel alkaloids and other phytochemicals with traditional and pharmacological uses. Amaryllidaceae biomolecules exhibit multiple pharmacological activities such as antioxidant, antimicrobial, and immunomodulatory effects. Traditionally, natural products from Amaryllidaceae are utilized to treat non-communicable and infectious human diseases. Galanthamine, a drug from this family, is clinically relevant in treating the neurocognitive disorder, Alzheimer’s disease, which underscores the importance of the Amaryllidaceae alkaloids. Although Amaryllidaceae provide a plethora of biologically active compounds, there is tardiness in their development into clinically pliable medicines. Other genera, including *Cyrtanthus* and *Tulbaghia*, have received little attention as potential sources of promising drug candidates. Given the reciprocal relationship of the increasing burden of human diseases and limited availability of medicinal therapies, more rapid drug discovery and development are desirable. To expedite clinically relevant drug development, we present here evidence on bioactive compounds from the genera *Allium*, *Tulgbaghia*, *Cyrtanthus* and *Crinum* and describe their traditional and pharmacological applications.

## 1. Introduction

Amaryllidaceae belongs to the order Asparagales and consists of bulbous flowering plants separated into three infrageneric ranks: Agapanthoideae, Allioideae and Amaryllidoideae, as delineated by the Angiosperm Phylogeny Group [1]. The term “Amaryllidaceae” is frequently used in either phytochemical or pharmacological literature to refer to plants or alkaloids originating from the subfamily Amaryllidoideae [2,3]. Monocotyledonous plants constitute seventy-nine genera (including *Allium*, *Crinum*, *Cyrtanthus*, and *Tulbaghia*) with over 1000 species [4]. Aside from their broad pantropical distribution, Amaryllidaceae are located in Africa, the Mediterranean Coast and South America, and have high adaptation and speciation [5]. The genus *Allium* is distributed in temperate, arid, semi-arid and subtropical areas such as the Mediterranean region, central Asia, Africa and parts of Europe. As herbaceous geophyte perennials, *Allium* comprises a plethora of species with pungent linear leaves that may or may not arise from a bulb or rhizome [6,7]. The *Tulbaghia* genus, popularly called “sweet garlic”, “wild garlic”, or “pink agapanthus”, is crown shaped with outgrowth or appendages of the perianth and predominantly colonizes the Eastern cape belt of South Africa, and is adapted for growth in areas such as Europe and America [8,9]. The genus *Crinum* encompasses 104 species and appear as showy flowers on leafless stems, which thrive in the tropics and warm temperate parts, specifically Asia, Africa, America, and Australia [10]. *Cyrtanthus* is popularly known as “fire lily” due to its unique rapidly flowering response to natural bush fires. Most species are found in South Africa and play an important role in South African traditional medicine [11].

Amaryllidaceous plants are known for their ornamental, nutritional, and medicinal value. Given their attractive flowering plant-like features, *Crinum* species are prized for their umbel lily-like blossoms in China and Japan [3]. Concurrently, Amaryllidaceae are known for their longstanding exploitation in medicinal therapy owing to their inherent biosynthesis of chemically diverse bioactive compounds with peculiar biological properties. The use of proximate and mineral composition analysis enabled the identification of phytoconstituents [10,12], while in vitro, in vivo, and in silico model systems have permitted the unravelling of intrinsic pharmacological activities of the natural products and other alkaloids isolated from this source [13,14,15]. Of note, bioactive compounds from Amaryllidaceae possesses a wide range of bioactivities ranging from antioxidant [16,17], anti-inflammatory [16,18], antimicrobial [17], antifungal [19], antiviral [20,21], antiplasmodial [22,23,24], anticarcinogenic [18,25,26], antispasmodic [1,27], antiplatelet [28], antiasthmatic [29], antithrombotic [30,31], antitumor [25], antihyperlipidemic [25], antihyperglycemic [25,32,33], antiarthritic [25], antimutagenic [16], immunomodulatory [16] and several others [34].

Given the aforementioned biological activities, *Allium*, *Tulbaghia*, *Cyrtanthus* and *Crinum* are utilized in traditional medicinal therapy for varying diseases and conditions [35,36,37,38,39,40,41]. For example, *Allium* is used as concoctions, decoctions, extracts, and herbal preparations to treat angina, amoebic dysentery, arthritis, cardiovascular diseases, cholera, catarrh, dysmenorrhea, fever, headaches, hepatitis, stomach disorders, throat infections, and prostatic hypertrophy [30,31,35,36,37,38]. The genus *Tulbaghia* has unique pharmacotherapeutic properties and is utilized to manage ailments such as earache, pyrexia, tuberculosis, and rheumatism [9,42]. *Crinum* species are used to treat haemorrhoids, malaria, osteoarthritis, varicosities, wounds, urinary tract infections, and gynaecological remedies [40,41]. *Cyrtanthus* are also employed in the management of ailments associated with pregnancy, as well as cystitis, age-related dementia, leprosy, scrofula, headaches, chronic coughs, among others [43,44]. In modern clinical practice, galanthamine from Amaryllidaceae is a primary choice of drug in managing symptomatic neurological disorders such as Alzheimer’s disease due to its selective inhibitory action on the acetylcholine biosynthetic enzyme, acetylcholinesterase [45]. The pancratistatin phenanthridone class of alkaloids are also promising chemotherapeutic drug candidates with unique cell line-specific antiproliferative properties, conferring a selective advantage for clinical development [46].

Although Amaryllidaceae represents a source of valuable bioactive compounds, developing promising drug candidates into clinically relevant therapeutics has been slow. Similarly, other genera in this family, including *Cyrtanthus*, *Crinum* and *Tulbaghia*, are untapped reservoirs and could serve as an alternative window for novel drug targets and warrant further investigation. This review consolidates evidence on the bioactive compounds from *Allium*, *Tulbaghia*, *Crinum* and *Cyrtanthus* and ascertains their traditional and pharmacological applications. Specifically, bibliographic searches were conducted on multiple standard databases (such as, Scopus, Web of Science, MEDLINE, Sci verse, Embase, Google scholar among others) using MESH and non-MESH terms to retrieve and synthesize relevant publications over the 3-month search period. This review highlights panoply of promising biomolecules from the taxa Amaryllidaceae and their prominent medicinal values. The evidence from this study could hasten drug discovery among the pharmaceutical industries. An update on the natural products from these lesser explored genera could also augment the lean pipeline of novel therapeutics.

## 2. The Genus *Tulbaghia*

### 2.1. Botanical Description

*Tulbaghia* is made up of monocotyledonous species with herbaceous perennial bulbs covered by brown scales and are mostly found in Africa [8]. South African species possess bulb-like corms or rhizomes which are swollen, irregularly shaped and wrapped in dry, fibrous leaves [8]. Members of this genus usually possess a raised crown-like structure or ring at the center of their flower tube [8]. Their seeds are black, flat and elongated with the mature ones having embryos [8]. Examples of species of this genus are *Tulbaghia violacea* (*T. violacea*), *Tulbaghia acutiloba* Harv. (*T. acutiloba*), *Tulbaghia capensis* L. (*T. capensis*) and *Tulbaghia cepacea* L.f (*T. cepacea*) [8].

### 2.2. Geographical Distribution and Traditional Uses of Tulbaghia Species

With approximately 66 species (https://www.kew.org/science accessed on 22 February 2022) [47], *Tulbaghia* is the second-most species-rich genus within Amaryllidaceae. *Tulbaghia* is a monocotyledonous genus comprised morphologically of herbaceous perennial bulbous species, which produce a variety of volatile sulfur compounds, hence resulting in a distinct pungent garlic odor released by bruised plants [8,48]. The genus was named by Carl Linnaeus after Ryk Tulbagh (1699–1771), a former governor of the Cape of Good Hope in South Africa, where most of the native species are to be found, particularly in the Eastern Cape Province [49]. In addition to South Africa, the genus is widely distributed across southern African countries including Botswana, Lesotho, Swaziland, and Zimbabwe, where the plant is revered in folk medicine being used for the treatment of a plethora of infectious and non-infectious diseases [9] as highlighted in Table 1.

### 2.3. Phytochemistry of Tulbaghia

**Figure 1:**
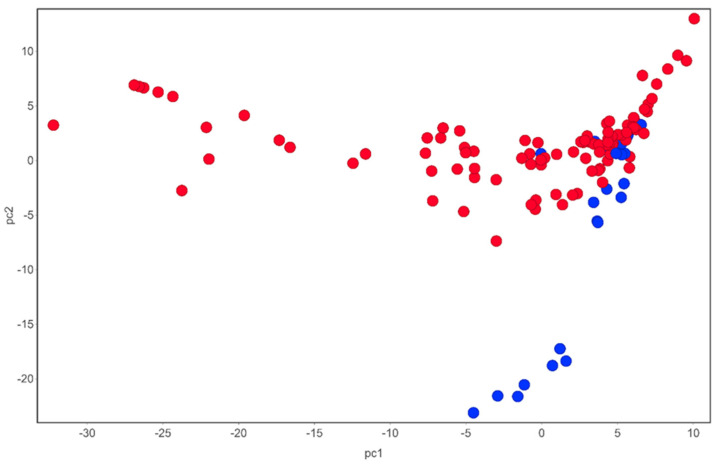
Chemical space of compounds identified from *T. violacea*. Blue circles are sulfur-containing compounds while red circles are compounds devoid of sulfur in their chemical structures. PCA analysis carried out using DataWarrior [54].

*Tulbaghia* produces many different classes of compounds with diverse chemical structures dominated by sulfur-containing natural products (Figure 1; Table S1).

**Figure 2:**
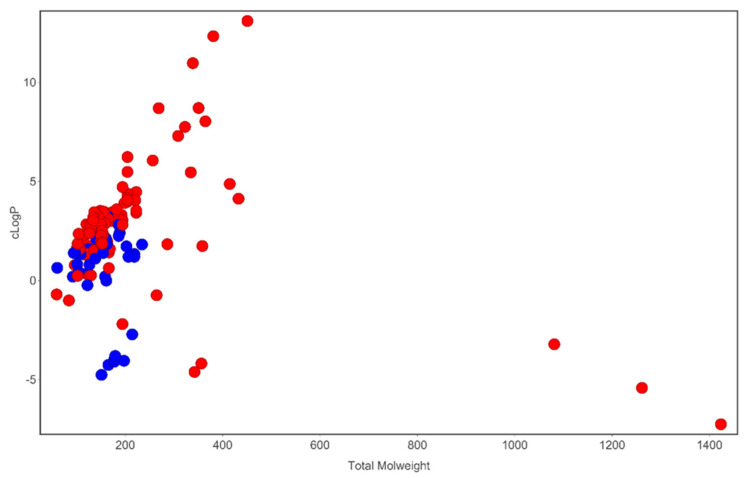
Analysis of cLogP and molecular weight space occupied by compounds identified in *T. violacea*. Blue circles are sulfur-containing compounds while red circles are compounds devoid of sulfur in their chemical structures. Plot generated using DataWarrior [54].

Most compounds reported have a small molecular weight (<500) and are of a broad lipophilicity (Figure 2).

**Figure 3:**
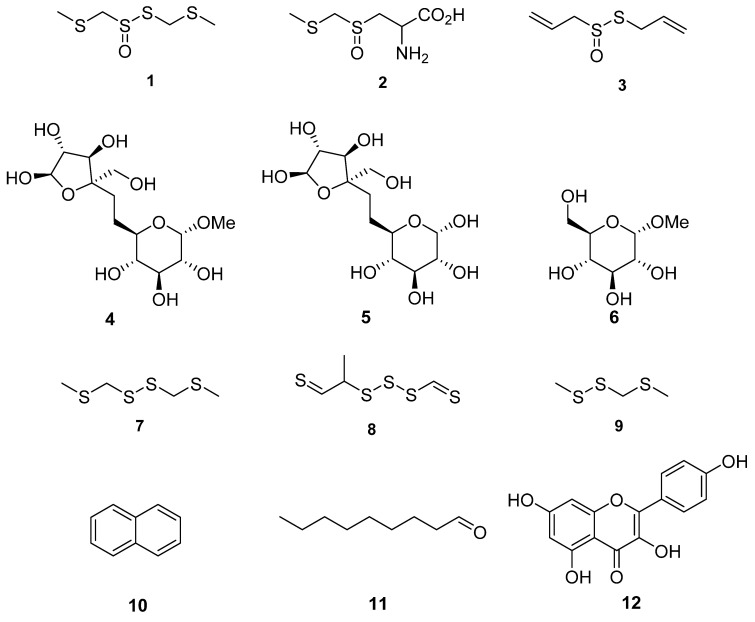
Chemical structures of compounds identified in *T. violacea*. (1) Marasmicin (**1**), (2) marasmin (**2**), allicin (**3**)—possesses antibacterial and antifungal activity, d-fructofuranosyl-β(2→6)-methyl-α-d-glucopyranoside (**4**), β-d-fructofuranosyl-(2→6)-α-d-glucopyranoside (**5**), methyl-α-d-glucopyranoside (**6**), bis(methylthiomethyl) disulfide (**7**)—found to constitute 48% of volatiles in aerial parts of *T. violacea* [55], methyl-2-thioethyl thiomethyl trisulfide (**8**)—found to constitute 16% of volatile compounds in aerial parts of *T. violacea* [55], methyl (methylthio)methyl disulfide (**9**)—found to constitute 10 % of volatile compounds in aerial parts of *T. violacea* [55], naphthalene (**10**)—interestingly observed to significantly increase in concentration in plants infected by the fungus *Beauveria bassiana* in comparison to untreated controls [59], nonanal (**11**)—also observed to significantly decrease in concentration in plants infected by the fungi *Beauveria bassiana* in comparison to untreated controls [59] and finally kaempferol (**12**)—which possesses multiple biological activities including antioxidant, anticancer and anti-inflammatory properties [60,61,62].

*Tulbaghia violacea* has been the most widely investigated for its phytochemistry and pharmacological properties. To date, close to 100 compounds have been tentatively identified, largely using gas chromatography techniques, from different parts of this species (Supplementary File S1) [55]. Most prominent are the sulfur compounds with reported broad-spectrum pharmacological activity. The thiosulfinate marasmicin (**1**) is the most prolific antimicrobial compound reported thus far from this genus [56]. This compound is formed from its precursor compound marasmin (**2**), by the enzyme c-lyase. Marasmicin is responsible for the characteristic garlic odor generated by damaged plants [48]. Other notable compounds produced by this species include phenols, tannins and flavonoids [55], which are also responsible for several observed biological activities. Phytochemical characterization has been carried out, albeit minimally for other *Tulbaghia* species particularly *T. alliacea* and *T. acutiloba*. Unlike other genera in Amaryllidaceae, *Tulbaghia* is so far devoid of any alkaloids [57,58]. Despite the extensive in vitro pharmacological screening of extracts of *Tulbaghia*, it is possible that less effort has been made to isolate and identify their active principles. Hence, the phytochemistry of the genus *Tulbaghia* largely remains understudied. The chemicals structurers of noteworthy compounds isolated from *T. violacea* have been represented in Figure 3.

### 2.4. Pharmacological Studies of Tulbaghia Species

Because of its perceived medicinal value, *Tulbaghia* has received marked interest within the scientific community which has meticulously subjected it to various in vitro and in vivo studies experimentally evaluating its pharmacological activities. The volume of published studies generated from these investigations mirror the distribution of the genus with most articles on *Tulbaghia* having emerged from South Africa (Table 2), a country highly rich in this genus both in terms of species diversity and abundance.

The greatest numbers of pharmacological screens have been on interrogating the antimicrobial properties of this genus. This is closely followed by cardiovascular, antioxidants and cancer investigations as shown in Table 3. *T. violacea* prominently features, being the most studied species, with *T. alliacea* and *T. aticulata* having received minimal attention.

#### 2.4.1. Antimicrobial and Antiparasitic Activity

As antimicrobial resistance continues to be a global health threat, the need to find therapeutic alternatives has never been more urgent [63]. This has encouraged scientists to search for novel alternatives with natural products having drawn marked interest as a potential oasis of new antimicrobial agents [64,65,66]. *Tulbaghia* has received significant relevance in this regard, with multiple studies providing ample evidence substantiating its use as an antimicrobial agent. Extracts of *T. violacea* have potency against many microbial species including those designated as priority by the World Health Organization. These include *Pseudomonas aeruginosa* (*P. aeruginosa*), *Staphylococcus aureus* (*S. aureus*) and *Klebsiella pneumoniae* (*K. pneumoniae*) with MIC values ranging between 20 and 300 µg/mL [67]. This activity was confirmed in another study where the disc diffusion method was used [68]. In addition to bacteriostatic activity, extracts of *T. violacea* have shown noteworthy potency against yeasts including *Candida albicans* (*C. albicans*) and *Candida parapsilosis* (*C. parapsilosis*) with MIC and MMC values ranging between 20 and 40 µg/mL [68]. Beyond human pathogens, extracts of *T. violacea* have activity against microorganisms of agricultural significance, for example against the fungus *Aspergillus flavus* (*A. flavus)*, which is responsible for significant agricultural produce loss at a global scale due to production of aflatoxins [69]. Extracts of *T. violacea* compromised cell wall synthesis by significantly reducing β-glucan and chitin synthesis in *A. flavus* corresponding to a dose-dependent inhibition of the enzymes β-glucan and chitin synthase, respectively [70]. Further studies suggested an alternative mode of action (MoA) via reduction of ergosterol production in fungi [71]. Interestingly, related to value in agriculture, a patent has been filed on the use of extracts of *T. violacea* as a plant protecting remedy as a substitute for chemical agents [72]. Some thought-provoking studies have shown that growth conditions including light intensities, watering frequency and pH, substantially impact both growth and biological potency of *T. violacea* extracts against *Fusarium oxysporum* (*F. oxysporum*) [73,74]. Likewise, storage conditions of dried plant material also affect the antimicrobial potency of extracts [56]. In addition to antimicrobial activity, *T. violacea* has shown good antiparasitic activity against the parasitic worm *Meloidogyne incognita* (*M. incognita*) on tomato roots and in soil [75]. Antiparasitic activity has also been observed against *Trypanosoma brucei* (*T. brucei*) (*IC*_50_ = 2.83 µg/mL) and *Leishmania tarentolae* (*L. tarentolae*) (*IC*_50_ = 6.29 µg/mL) [67]. Table 4 highlights the antimicrobial activity of *Tulbaghia* species.

#### 2.4.2. Anticancer Activity

Owing to the need for novel anticancer agents [76] and motivated by the success of cancer drug discovery projects from natural products [77], Mthembu and Motadi in (2014), evaluated the in vitro anticancer properties of crude methanol extracts of *T. violacea* using an MTT assay [78]. Extracts displayed time- and concentration-dependent antiproliferative properties against cervical cancer cell lines with an *IC*_50_ of 150 µg/mL. The MoA was deciphered to be induction of apoptosis by a p53-independent pathway [78]. However, in contrast to this finding, continued work showed a proportional increase in the activity of caspase 3/7, and the expression of p53 genes strongly suggests apoptosis was triggered by a p53-dependent pathway [79]. This latter finding has been partly substantiated by data emerging from a study examining the antineoplastic properties of *T. violacea* against ovarian tumor cells. These extracts were shown to partially induce both apoptosis and necrosis with the most pronounced activity due to induction of autophagy [80].

Triple-negative breast cancer remains one of the most challenging cancers, being highly aggressive [81]. *T. violacea* extracts have demonstrated good cytotoxic activity against MDA-MB-231, with an *IC*_50_ of 300 µg/mL [82]. Additionally, extracts inhibited migration of the cancer cell lines (metastasis), an important physiological process in the progression of this cancer [83]. In addition to the gynecological cancers, antineoplastic properties of *T. violacea* were further observed against pancreatic cancer with 63% inhibition of cell proliferation at a concentration of 250 µg/mL [68]. Against a non-sex-specific cancer, *T. violacea* showed noticeable activity against oral cancer with an *IC*_50_ of 0.2 and 1 mg/mL for acetone and water-soluble extracts, respectively. Extracts activated caspase activity in a dose-dependent manner leading to induction of apoptosis in the human oral cancer cell line [84]. Using a bioassay guided approach, the active anticancer compounds in *T. violacea* have been identified to be glucopyranosides d-fructofuranosyl-β (2→6)-methyl-α-d-glucopyranoside and β-d-fructofuranosyl-(2→6)-α-d-glucopyranoside. Both compounds act by mediating induction of apoptosis in Chinese hamster cells by targeting the mitochondrial (intrinsic) pathway [85,86]. A summary of the anticancer activity of *Tulbaghia* species is shown in Table 5.

#### 2.4.3. Antioxidant Activity

The imbalance of reactive oxygen species (ROS) and antioxidants in the body can lead to oxidative stress [87]. This physiological condition can result in cellular and tissue damage [88]. Oxidative stress is associated with pathologies including cancer, cardiovascular disease, diabetes, and neurodegenerative diseases amongst others [88,89]. To avert the development of oxidative stress, attenuation of ROS has been identified as a viable target, with natural products seen as a potential source capable of neutralizing it [88]. *Tulbaghia* has generated some interest on this front particularly as it is rich in compounds with proven antioxidant activity including phenols, tannins and flavonoids. Multiple studies have demonstrated that extracts of *Tulbaghia* have marked antioxidant activity as assessed using different assays in vitro including Trolox equivalent antioxidant capacity (TEAC; also commonly referred to as the ABTS assay), ferric-reducing antioxidant power (FRAP) and 2,2-diphenyl-1-picryl-hydrazyl-hydrate (DPPH) (Table 6) [58,80,90,91]. Furthermore, using an in vivo model of *Caenorhabditis elegans*, *T. violacea* extracts attenuated oxidative stress produced by a free radical generator, (2,2′-azobis-2-amidinopropane dihydrochloride; AAPH), in the roundworm [80]. Data from these studies strongly suggested continued investigation of other species in the search for more potent antioxidant agents from *Tulbaghia*. The antioxidant activity of *Tulbaghia* species is highlighted in Table 6.

#### 2.4.4. Antidiabetic, Anticardiovascular and Antithrombogenic Activity

The incidence of diabetes and cardiovascular diseases continues to grow substantially across the globe, with both conditions combined accounting for the highest global morbidity and mortality [93,94]. Both of these chronic conditions are closely linked with cardiovascular disease being responsible for high morbidity and mortality in diabetic patients [95]. *Tulbaghia* has been documented in ethnopharmacological studies for the treatment of these ailments with emerging scientific data strongly validating its use. In streptozotocin diabetes-induced rat models, *T. violacea* attenuated diabetes-associated physiological conditions resulting in improved body weights, reduced fasting blood glucose levels, enhanced glucose tolerance and significantly elevated plasma insulin and liver glycogen content [96]. These data were corroborated in another study in which *T. violacea* noticeably reduced blood glucose and serum lipid (triglyceride (TG), total cholesterol (TC), and very low-density lipoprotein (VLDL)) levels while raising plasma insulin in a streptozotocin-induced diabetic rat model [97]. In an assessment for negating cardiovascular associated conditions, *T. violacea* in in vivo models markedly reduced systolic blood pressure (BP), diastolic BP, mean arterial pressure (MAP) and the heart rate in both age-induced and spontaneous hypertensive rats [98]. Furthermore, dosing rats with extracts of *T. violacea* led to improved kidney function [99]. This is an essential pharmacological property as kidney function is impaired in hypertension leading to high morbidity and mortality in people suffering from cardiovascular diseases [100].

One of the multiple factors strongly associated with cardiovascular disease is atherothrombotic vascular disease (AVD). Platelet aggregation plays a role in development of AVD and subsequent cardiovascular events [90,101]. Against this background, platelet aggregation has been identified as a key process to target to prevent AVD. Encouragingly, *T. violacea* demonstrated marked potency being able to significantly inhibit platelet adhesion 15 min post-exposure (Table 7) [90,92].

#### 2.4.5. Miscellaneous Pharmacological Activity

In addition to diabetes and cardiovascular diseases, *T. violacea* has shown activity against another chronic condition, Alzheimer’s disease. In an in vivo Alzheimer’s disease transgenic *C. elegans* strain model, *T. violacea* significantly reduced 1-42 β-amyloid peptide formation (Table 8) [80]. *T. violacea* exhibited in vivo anticonvulsant activity by attenuating tonic convulsions induced by either pentylenetetrazole, bicuculline, picrotoxin, strychnine or NMDLA [102] and validating its traditional use for the treatment of epilepsy. *T. violacea* displayed marked tick repellence properties of fungus-exposed plants at low treatment concentrations (5% *w*/*v* and 10% *w/v*) [59], further enhancing its credentials as a potential agricultural product. Somewhat concerning is that, extracts of *T. violacea* also induced genotoxic effects albeit at high test concentrations (250, 500 and 1000 µg/mL) in the *Allium cepa* assay [103]. Furthermore, broad murine macrophage antiproliferative and cytotoxicity activity, influenced by extract test concentrations, type of solvent and plant part used, have been observed (Table 8) [104]. There is consequently a need for rigorous assessment of safety of extracts of this and other species of the genus *Tulbaghia*.

## 3. The Genus *Allium*

### 3.1. Botanical Description

Species of the genus *Allium* are mostly found in warm–temperate and temperate zones of northern hemisphere as well as the boreal zone [105]. They are petaloid perennial herbs with parallel narrow leaves [33] and possess true bulbs, which are sometimes found on rhizomes [106]. *Allium* species are also characterized by onion or garlic odor and flavor similar to *Tulbaghia* [106]. Well known species include *Allium cepa* (*A. cepa*), *Allium sativum* (*A. sativum*), *Allium ascalonicum* (*A. ascalonicum*,), *Allium porrum* (*A. porrum*), and *Allium schoenoprasum* (*A. schoenoprasum*) (chive) [33]

*Allium* has over 500 species making it the largest genus of Amaryllidaceae [6,7]. There are plethora of species, notably *A. cepa* and *A. sativum* [107,108,109]. Other examples grown for their medicinal and nutraceutical value are *Allium ducissae* (*A. ducissae*), *Allium strictum* (*A. strictum)*, *Allium umbilicatum* (*A. umbilicatum*), *Allium victorialis* (*A. victorialis*), *A. ascalonicum*, *Allium chinense* (*A. chinense*), *Allium tuberosum* (*A. tuberosum*), *Allium griffithianum* (*A. griffithianum*), *Allium oreoprasum* (*A. oreoprasum*), and *Allium oschaninii* (*A. oschaninii*). Species tolerate varying climatic conditions, hence are geographically distributed across several continents, including Asia, Africa, the Americas, and Europe [107,110]. Fernandes et al. identified *A. cepa* that colonizes four different geographical regions of the Madeira island, an archipelago near the North Atlantic ocean with a hot and/or warm-summer Mediterranean climate conditions [107]. As the world’s second-most relevant and cultivated horticulture vegetable crop, the onion (*A. cepa*), is distributed in over 175 countries and covers approximately six million hectares of the total land size of the world. Approximately two-thirds (66%) of global onion production emanates from the Asia, with China and India being the world’s largest producers [111]. The maximal diversification of *A. cepa* is found in Iran and Afghanistan’s Mediterranean basin. *A. cepa* thrives in areas with boreal, temperate, and tropical climates [108]. Similarly, *A. sativum* (garlic) bears close resemblance to onions and originates from Central Asia but has spread to include regions in Europe, America, and Africa [112]. The global garlic production estimates show that out of the 28.5 million tonnes (MT) of *A. sativum* cultivated, the majority (91.6%; 26.1 MT) were from Asia, followed by Europe (3.0%; 0.86 MT), America (2.9%; 0.83 MT), and with the least from Africa (2.7%; 0.73 MT) [112]. Bartolucci et al. identified *A. ducissae*, a new breed of *Allium* that grows in the mountainous regions of the Central Apennines in the Abruzzo and Lazio counties of Italy [113]. Furthermore, *A. strictum*, a Eurasian species, is distributed across China, Europe, Russia, Kazakhstan, Kyrgyzstan, and Mongolia [114,115]. *A. umbilicatum*, also called gladiolus or leek is usually localized in semi-arid regions and can tolerate sub-zero freezing winters [116]. It occurs as a weed in oases and span across Afghanistan, Iran, Pakistan, Turkmenistan, Tajikistan, and central and Eastern Asian regions [116]. As a representative circumboreal plant, *A. victorialis* has a wide altitudinal climatic tolerance [117]. It is predominantly located in lowland deciduous forest and subalpine birch forest, but seldom found in the subalpine meadows [117]. This species is scattered distribution on the island stretches of Japan, Russia, and Northern China [117,118]. Although practically grown throughout the world, *A*. *ascalonicum*, also called shallot, is native to the Middle East, and the name is derived from the Syrian city Ascalon. These shallots are distributed on the main islands of Indonesia, in Bangladesh, Japan, Korea, Malaysia, Taiwan, and Thailand [119]. *A. chinense* (locally referred to as Chinese/Japan onion or scallion, Kiangski scallion, oriental onion, Rakkyo) is an uncommon *Allium* species found mainly in the tropical and sub-tropical regions of China, Japan, Vietnam, and eastern areas of India [111,120]. *A. tuberosum* is an indigenous species native to southeastern Asia and regarded as a late-seasonal bloomer. During the initial growth phases, *A. tuberosum* is evergreen in hot climates but succumbs to cold climatic conditions. However, the Chinese chive becomes tolerant to all seasonal variations [121,122]. *A. griffithianum* and *A. oreoprasum* are geographically skewed towards the mountainous regions of Pakistan, Afghanistan, Kyrgyzstan, Uzbekistan, and Tajikistan [123], whereas *A. oschaninii* are located in the Darvaz mountains of Central Tajikistan [124].

### 3.2. Traditional Uses of Genus Allium

Increasing scientific evidence asserts the traditional uses of plants in folklore medicine [124,125,126]. Researchers over the years have investigated various parts of local medicinal plants to identify phytoconstituents with potential bioactivity, and further develop them into new drug therapies [127,128]. *Allium* species contain the common phytocompounds (anthocyanins, flavonoids, organosulfur, sterols, saponins, phenolic acids, amino acids, vitamins and minerals) [129,130,131,132] with innumerable biological properties [130,131,132,133]. Owing to these biological advantages, *Allium* species are locally used in managing various diseases affecting human organs and organ systems such as inflammation, microbial pathologies and oxidative stress injuries [130,131,132,133]. In particular, *A. cepa* is used to treat alopecia, hearing impairment, menstrual disorders, erectile dysfunction and ocular and metabolic diseases [133,134,135]. Similarly, *A. sativum* is employed in the management of hematological disorders, carcinomas, muscle weakness and compromised airways [135,136,137,138,139]. Other varieties of *Allium* species also serve as appetizers, nerve soothers, and relieving agents against digestive, respiratory, and urinary system discomfort as seen in Table 9.

### 3.3. Phytochemistry of Allium

Owing to the numerous traditional uses of these species, it is not surprising that the genus contains several phytoconstituents which may be responsible for their observed activity. Table 10 outlines various phytochemicals isolated, their geographic location and their biological activity.

**Figure 4:**
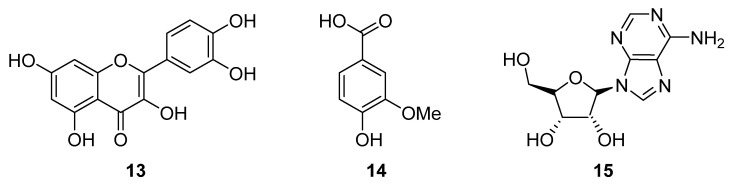
Chemical structures of compounds isolated from the genus *Allium*. Quercetin (**13**), vanillic acid (**14**) and adenosine (**15**).

The chemical structures of compounds from the genus *Allium* are shown in Figure 4.

### 3.4. Pharmacological Effects of Allium

There are several species within *Allium* whose biological activities have been well established [198]. This section focuses on the pharmacological activities associated with these species.

#### 3.4.1. Antimicrobial Activities

Garlic has shown antimicrobial effects against Gram-positive, Gram-negative and acid fast stain organisms [199,200,201]. Allicin from garlic showed effectiveness toward methicillin-resistant *S. aureus* (MRSA) [200]. Extracts from garlic also showed broad-spectrum fungicidal effect against several fungi including *Candida*, *Trichophyton*, *Cryptococcus*, *Aspergillus*, *Trichosporon* and *Rhodotorula* species. Garlic extract was recently found to inhibit *Meyerozyma guilliermondii* and *Rhodotorula mucilaginosa* germination and growth [202]. A study by Fufa reported the antifungal activity of various *A. sativum* extracts, namely aqueous, ethanol, methanol, and petroleum ether against human pathogenic fungi such are *Trichophyton verrucosum*, *T. mentagrophytes*, *T. rubrum*, *Botrytis cinereal* (*B. cinerea)*, *Candida species*, *Epidermophyton floccosum*, *A. niger*, *A. flavus*, *Rhizopus stolonifera*, *Microsporum gypseum*, *M. audouinii*, *Alternaria alternate*, *Neofabraea alba*, and *Penicillium expansum* [203]. Essential oil from garlic showed antifungal activity against a number of fungi such as (*C. albicans*, *C. tropicalis* and *Blastoschizomyces capitatus*). Saponins extracted from *A. sativum* had antifungal activity against *B. cinerea* and *Trichoderma harzianum* [204]. *Allium* species from Ghana were reported by Danquah et al. to possess anti-infective and resistance modulatory effects on selected microbial strains [205]. *Allium hirtifolium* was found to exhibit antimicrobial activities against *E. faecalis* [206].

Previous studies have shown that garlic extract inhibit the growth of *Blastocystis* species in vivo and this effect was attributed to the several phytochemicals contained in garlic extracts. Examples of these phytochemicals are thiosulfinates and allicin which have been investigated to possess antibacterial and antiprotozoal effects [204,207]. Garlic extracts have been evaluated for antiviral effects against influenza B, human rhinovirus type 2, human cytomegalovirus (HCMV), parainfluenza virus type 3, *Herpes simplex* type 1 and -2, vaccinia virus, and vesicular stomatitis virus [208]. Danquah et al. again reported the antitubercular effects of analogues of disulfides from *A. stipitatum* as well as their anti-biofilm and anti-efflux effects [209].

#### 3.4.2. Antioxidant Properties

It has been reported that frequent garlic intake promotes internal antioxidant activities and reduces oxidative adverse effects either by increasing the endogenous antioxidant synthesis or reducing the production of oxidizing agents such as oxygen-free radical species (ORS) [210]. It has also been demonstrated that garlic possesses protective properties against gentamycin as well as acetaminophen-induced hepatotoxicity by improving antioxidant status, and regulating oxidative stress [200]. Garlic extract was found to elevate the activities of selected antioxidant enzymes (e.g., superoxide dismutase (SOD)) and decrease glutathione peroxidase (GSH-Px) in rats’ hepatic tissues [13,118,211]. Saponins extracted from garlic were reported to scavenge intracellular ROS and protect mouse-derived C_2_C_12_ myoblasts towards growth inhibition and H_2_O_2_-induced DNA damage [13,212]. *A. ursinum* aqueous extract also demonstrated antioxidant effect which lasted approximately 16 h [213]. *A. hirtifolium* was reported to possess antioxidant capacity by neutralizing the free radical species in a system [214].

#### 3.4.3. Anti-Inflammatory Properties

It has been reported widely that garlic extracts and its related phytochemicals possess anti-inflammatory activity. A study by Ahmad et al. revealed that garlic extracts significantly impaired liver inflammation and damage caused by *Eimeria papillata* infections [215]. The mechanism underlying the anti-inflammatory effects of garlic was attributed to the inhibition of emigration of neutrophilic granulocytes into epithelia as described by Hobauer et al. [216] and Gu et al. [217]. The chloroform extract of aged black garlic acts by reducing NF-κB activation in human umbilical vein endothelial cells caused by tumor necrosis factor-α (TNF-α) and the methanolic extract also reported to prevent the cyclooxygenase-2 (COX-2) and prostaglandin E_2_ (PGE_2_) production by NF-κB inactivation [218]. A report by Jin et al. confirmed that thiacremonone (a sulfur compound isolated from garlic) prevents neuroinflammation and amyloidogenesis by blocking the NF-κB activity, and therefore makes it an ideal remedy to manage neurodegenerative disorders (e.g., Alzheimer’s disease) related to inflammation [219].

Krejčová et al. reported that pyrithione and related sulfur-containing pyridine *N*-oxides from Persian shallot possessed anti-inflammatory and neurological activity [220]. The extracts of *A. stipitatum* were reported to exhibit antibacterial effect in vivo against methicillin-resistant *S. aureus* [221]. Anti-inflammatory effect of *A. hookeri* on carrageenan-induced air pouch mouse model was also established by Kim et al. [222].

#### 3.4.4. Anticancer Activity

Comparison of the anticancer effect of raw garlic extracts against other extracts from different plants found garlic to be the most effective and highly specific anticancer agent [223]. The anticancer mechanisms of garlic extracts were reported to be mediated via inhibition of cell growth and proliferation, regulation of carcinogen metabolism, stimulation of apoptosis, prevention of angiogenesis, invasion, and migration; and thus affording the anticancer agent with minimal negative effects [13]. Chabria et al. reported that allicin isolated from garlic suppresses colorectal cancer metastasis through enhancing immune function and preventing the formation of tumor vessels as well as surviving gene expression to enhance the cancer cell’s apoptosis [224]. Fleischauer and Arab [225] reported that continuous garlic intake could decrease different kinds of cancer propagation such as cancer of the lung, colon, stomach, breast, and prostate. Piscitelli et al. reported that garlic reduced the plasma concentrations of saquinavir by approximately 50% in healthy participants after a 3-week garlic supplement intake. In addition to this, many researchers evaluated the antitumor and cytotoxic actions of garlic and its related constituents in vitro and in vivo [226].

#### 3.4.5. Other Pharmacological Effects of Allium Species

Investigations on extracts of *A. sativum* (garlic) revealed anticholinesterase effects, which could be further developed and utilized in the management of Alzheimer’s disease [227,228,229]. Garlic is known to possess hypolipidemic effects by reducing the total glycosaminoglycans concentration in heart and aorta [230]. Garlic is also known to reduce the level of cholesterol either by acid stimulation and excretion of neutral steroids or by reducing the cholesterogenic and lipogenic effects of fatty acid synthase, 3-hydroxy-3-methyl-glutaryl-CoA reductase, malic acid, and glucose-6 phosphate dehydrogenase in hepatocytes [231]. Garlic tablets formulated by Ashraf et al. and administered at a dose of 600 mg/day for 12 weeks in diabetic patients with dyslipidemia resulted in high HDL, low LDL and TC levels [232].

Allicin, a constituent in garlic, was found to reduce diabetes mellitus in rats, which was similar to that demonstrated by glibenclamide and insulin [233]. Garlic extracts reduce body weight, adipose tissue mass and improved plasma lipid profiles in mice with high-fat diet-induced obesity [234]. The mechanism of these activities is downregulation of multiple gene expression such as adipogenesis along with upregulation of the mitochondrial inner membrane proteins expression [234]. Garlic extract is widely known to significantly control blood pressure by reducing both systolic and diastolic pressures [235]. Moreover, several reports have confirmed the antihypertensive effects of garlic [236]. Extracts of *A. stipitatum* were also assessed and established to possess significant wound healing properties [237].

## 4. The Genus *Crinum*

### 4.1. Geographical Distribution of Crinum

*Crinum*, which also belongs to the Amaryllidaceae family, comprises approximately 160 beautiful lilies that grow naturally in coastal areas of the tropics and subtropics. They are widely distributed in Africa, Asia, Australia and America [238,239,240,241]

### 4.2. Traditional Uses of Crinum

Plants of the genus *Crinum* have been used to treat various diseases across the world [242]. In China and Vietnam, *Crinum* plants in traditional medicine are believed to possess antiviral and immune-stimulatory properties. A hot aqueous extract of *Crinum latifolium* (*C. latifolium*) is used as an antitumor agent. *Crinum asiaticum* (*C. asiaticum)* is used in Malaysia to treat rheumatism and to relieve local pain [239]. *Crinum amabile* Donn. (*C. amabile*) is used in Vietnam to induce emesis, as well as for rheumatism and earache [241].

The bulbs of *C. asiaticum* L. are used as a tonic, laxative and expectorant in Indian traditional medicine, as well as for treating urinary tract diseases [241]. The seeds are used as purgatives, diuretics, and tonics, while the raw roots are used as an emetic. The leaves are also very useful in the management of skin problems, inflammation and cough [241]. *C. latifolium* L. is also used to treat rheumatism, abscesses, earaches, and as a tonic. *Crinum pratense* (*C. pratense)* and *Crinum longifolium* (*C. longifolium)* are also used as bitter tonics, laxatives and in the management of chest illnesses [243].

*Crinum zeylanicum* (*C. zeylanicum)* L. is used in Sri Lanka to treat abscesses and fevers; the bulbs are also used as rubefacient in rheumatism and against snake bites; and the juice from the leaves used to treat earaches [244].

The roots of *Crinum* species have been used in African traditional medicine to cure urinary infections, coughs and colds, renal and hepatic disorders, ulcers, sexually transmitted infections, and backache, as well as enhance breastfeeding in both animal and human mothers [241]. *Crinum kirkii* Bak. (*C*. *kirkii)*, a widespread East African grassland plant, is used to heal wounds in Kenya. In Tanzania, the fruit and inner part of the bulbs are used as purgatives, and the outer scales employed as rat poison [245,246]. Extracts of *Crinum delagoense* (*C. delagoense)* Verdoorn is utilized in Zulu and Xhosa traditional medicine in South Africa to treat urinary tract infections and body oedema [247,248,249]. Rheumatism, aching joints, septic sores, varicose veins, and kidney and bladder infections have all been treated using the South African *Crinum bulbispermum* (*C. bulbispermum)* [250]. In Cameroon, *Crinum pupurascens* (*C. pupurascens)* Herb is used to treat sexual asthenia and spleen disorders. *Crinum* species (*C. defixum* Keraudren et Gawl., *C. firmifolium* Baker, *C. modestum* Baker) are as well used in Madagascar to treat abscesses, anthrax, and otitis. It is also employed as an emetic, diaphoretic, and emollient. Externally, *Crinum firmifolium* (*C. firmifolium*) is used to treat a variety of parasite skin afflictions [40,243].

### 4.3. Phytochemistry of Crinum

Several phytochemical and pharmacological studies have been conducted on the genus *Crinum.* The compounds isolated from various species of *Crinum* as well as their biological activities have been outlined in Table 11.

**Figure 5:**
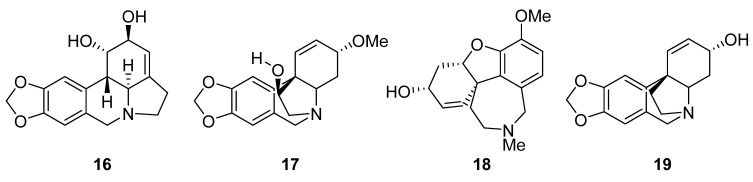
Chemical structures of compounds isolated from the genus *Crinum*. Lycorine (**16**), crinamine (**17**), galantamine (**18**) and crinine (**19**).

Chemical structures of common compounds from *Crinum* are shown in Figure 5.

### 4.4. Pharmacological Activities of Crinum

#### 4.4.1. Anti-Inflammatory and Analgesic Effects

The anti-inflammatory and the analgesic properties of various *Crinum* species have been investigated by several authors. The anti-inflammatory effect of *C. asiaticum* as well as its effect on bradykinin-induced contractions on isolated uterus has been reported [288,289,290,291]. The ethanolic extract of *C. asiaticum* demonstrated significant analgesic effect in an acetic-acid-induced writhing test [292]. Antipyretic and anti-inflammatory properties of *C. jagus* were recently reported by Minkah and Danquah [291]. Leaf extract of *C. bulbispermum* has also been established to possess antinociceptive effects [293,294].

#### 4.4.2. Anticancer and Cytotoxicity Effects

The cytotoxic effects of *C. asiaticum* extract was investigated and was shown to exert toxic effect on brine shrimps and murine P388 D1 cells [294,295,296,297,298]. Yui et al. demonstrated that hot water extracts of *C. asiaticum* exhibited potent inhibition of calprotectin-induced cytotoxicity in MM46 mouse mammary carcinoma cells. This activity whi1ch was later attributed to lycorine, an active compound in *C. asiaticum* [297]. Some alkaloids isolated from the bulbs of *C. asiaticum* have been reported to show remarkable inhibition against tumor cell lines A549, LOVO, HL-60, and 6T-CEM [261].

The extract of *C. asiaticum* exhibited antiproliferative and chemosensitizing effects against multi-drug-resistant cancer cells [298,299]. The antiangiogenic activity of the methanolic leaf extract of *C. asiaticum* was evaluated and established by Yusoff [300]. The cytotoxic effect of the essential oil extracted from *C. asiaticum* was as well established in MCF-7 cells [301]. A recent work done by Yu et al. reported the inhibition of the growth of HepG 2 cells in a dose-dependent manner by polysaccharide CAL-n, an isolate from *C. asiaticum* [262]. Also, the neuroprotection and anti-neuroinflammatory effects in Neuronal Cell Lines were reported by Lim et al. [279,302]. Alkaloids from *C. bulbispermum* have also been reported to possess cytotoxic activities [284]. Evaluation of the cytoprotective potential of *C. bulbispermum*, after induction of toxicity using rotenone, in SH-SY5Y neuroblastoma cells proved that, the plant has such effect as reported [303]. Aboul-Ela et al. [279] tested the cytotoxic effect of *C. bulbispermum* bulbs using the brine shrimp bioassay.

#### 4.4.3. Antimicrobial Properties

The in vitro antitubercular effects of *C. asiaticum* on *Mycobacterium tuberculosis* (*M. tuberculosis*) surrogate, *Mycobacterium smegmatis* (*M. smegmatis*), were reported [291,304]. *C. asiaticum* was shown to possess a broad-spectrum antimicrobial activity against Gram-positive, Gram-negative bacteria and fungal pathogens [291,299]. Antifungal activities of the essential oil and extracts of *C. asiaticum* against pathogenic fungi have also been established [305,306]. It is reported that the methanolic root extract of *C. asiaticum* exerts significant anti-HIV-1 activity [307]. The ethanolic extract of *C. asiaticum* significantly inhibited selected bacteria as evaluated by Naira et al. [308]. Dichloromethane extract of *C. asiaticum* was found to be the most effective against selected oral and vaginal *Candida* species [309]. Minkah and Danquah again demonstrated the antimicrobial activity of extracts of *C. jagus* against clinically significant microorganisms in the High-throughput spot culture growth inhibition (HT-SPOTi) assay [291]. Water/Ethanol extract of *C. jagus* was observed to be active on *Shigella flexneri*-induced diarrhea in rats [310]. The antimicrobial and antioxidant properties of *C. jagus* make it suitable as a wound healing agent [311]. The crude methanolic extract of *C. jagus* was investigated to have effect on *Mycobacterium tuberculosis* [312,313]. The crude alkaloid of *C. jagus* inhibited Dengue virus infection [314]. *C. macowanii* has also been shown to possess biological effects such as antifungal, antiviral and antiplasmodial activities [315].

#### 4.4.4. Antioxidant Properties

There antioxidant effects of *C. asiaticum* have been studied extensively. The ethanolic extract exhibited protective effects on human erythrocyte [316]. *C. asiaticum* bulbs also exerted remarkable free radical scavenging ability [317]. The antioxidant activity of the ethanolic extract of *C. asiaticum* leaves in alloxan-induced diabetic rats was well demonstrated [318]. More recent work on the methanolic extract of *C. asiaticum* showed antioxidant effects [319]. Potent DPPH radical scavenging activity was also observed for the aqueous *C. asiaticum* leaf extract [304]. Both the leaves and bulbs of *C. jagus* are important sources of antioxidant compounds [320]. A methanolic bulb extract of *C. bulbispermum* showed mild radical scavenging activity [321]. The leaf extracts of *C. bulbispermum* also showed modest antioxidant activity in a thiobarbituric acid reactive substances assay [297].

#### 4.4.5. Other Pharmacological Properties

Kumar reported the wound healing activities of the ethanolic *C. asiaticum* extract. The extract was found to possess pro-healing effects when topically applied on animal models by influencing various stages of healing process [322]. *C asiaticum* extract and norgalanthamine potentially influenced hair growth via inhibition of 5α-reductase activity and TGF-β1-induced canonical pathway [39,314]. There is a report on the inhibitory effects of three *C. asiaticum* genotypes against key enzymes implicated in the pathogenesis of Alzheimer’s disease and diabetes [319].

The anti-obesity effect of the *C. asiaticum* extract on a high-fat diet-induced obesity in monogenic mice has been reported [323,324]. An active fraction of *C. jagus* was shown to possess anticonvulsant activities in experimental rats [325].

Ethyl acetate and methanol extracts of *C. bulbispermum* have also been shown to exhibit acetylcholinesterase inhibitory properties [321]. The alkaloid galanthamine isolated from *C. bulbispermum* and other genera of Amaryllidaceae, has been approved for the treatment of Alzheimer’s disease [326]. Cognitive enhancing effect of a hydroethanolic extract of *C. macowanii* against memory impairment induced by aluminum chloride in balb/c mice has as well been reported [327].

## 5. The Genus *Cyrtanthus*

### 5.1. Botanical Description

Another large genus of the family Amaryllidaceae is *Cyrtanthus*. *Cyrtanthus* is derived from a Greek word for curved flower [6]. Species of this genus have numerous, black, winged seeds and give off a strong onion smell [6]. They possess a rhizome or bulb, flowers and a loculicidal capsule fruit [6]. They have leaves that are linear to lorate [6]. Flowers are funnel shaped with their stamens fixed in the corolla tube [6]. Species that belong to this genus include *Cyrtanthus elatus* (*C. elatus*) (Jacq.) Traub, *Cyrtanthus obliquus* (*C. obliquus*) (L.f.) Aiton, and *Cyrtanthus mackenii* (*C. mackenii*) Hook [44].

### 5.2. Geographical Distribution

*Cyrtanthus* is diverse and is a large sub-Saharan Africa genus consisting of approximately 55 species found mostly in South Africa. *Cyrtanthus* extends from the summer-dry southwest to the summer rainfall northeast [328]. The genus displays diverse floral morphology. The three major lineages show varying biogeographic affinities.

Clade A comprises taxa located in Southern African Grassland Biome with a few outliers in the Savanna Biome to the east and north, the Indian Ocean Coastal Belt Biome to the extreme east and the Fynbos Biome to the south [328]. Hence, it falls in the Afrotemperate Phytogeographical Region [329] that encompasses Afromontane phytochorion in the north and the Cape Floristic Region in the south [328]. Most existing species in the Afrotemperate lineage (*Cyrtanthus attenuatus* (*C. attenuatus)*, *Cyrtanthus macowanii* (*C. macowanii)*, *Cyrtanthus epiphyticus* (*C. epiphyticus)*, *C. mackenii* subsp. *cooperi*, *Cyrtanthus huttonii* (*C. huttonii)*, *Cyrtanthus macmasteri* (*C. macmasteri)*, *Cyrtanthus suaveolens* (*C. suaveolens)*, *Cyrtanthus stenanthus* (*C. stenanthus* var. *stenanthus*) and *Cyrtanthus flanaganii* (*C. flanaganii)* occur currently in the south-eastern African temperate grasslands. *Cyrtanthus tuckii* var. *transvaalensis* (*C. tuckii)* is the only species found in the grassland of the Highveld in the northern parts of South Africa. Few species are found outside this grassland area and includes *Cyrtanthus angustifolius* (*C. angustifolius)*, *Cyrtanthus fergusoniae* (*C. fergusoniae)* and *Cyrtanthus aureolinus* (*C. aureolinus)* in the Cape Region together with *C. mackenii* subsp. *Mackenii* and *Cyrtanthus brachyscyphus* (*C. brachyscyphus)* that occupies drainage lines on the subtropical Indian Ocean Coastal Belt [330]. Southern Africa is the area where *Cyrtanthus breviflorus* (*C. breviflorus)* is found extending northwards in a series of disjunct populations along mountain corridors to East Africa and Angola.

Clade B is limited to the Fynbos and Succulent Karoo Biomes which constitute the Greater Cape Region, referred to hereafter as ‘the Cape’ [331]. *Cyrtanthus labiatus* (*C. labiatus)* and *Cyrtanthus montanus* (*C. montanus)* from the Baviaansklo of Mountains and Eastern Cape are found at the interface of the Fynbos and Albany Thicket Biomes. The Richtersveld species, *Cyrtanthus herrei* (*C. herrei)* is found in the semi-arid Succulent Karoo [328]. Most species found in ‘the Cape’ lineage are located on the summer-dry, southeast coast forelands with half the number in the Fynbos of the nonseasonal rainfall Eastern Cape. *Cyrtanthus carneus* (*C. carneus*, *C. elatus*, *Cyrtanthus guthrieae* (*C. guthrieae*, *C. labiatus*, *Cyrtanthus leptosiphon* (*C. leptosiphon*), *Cyrtanthus leucanthus* (*C. leucanthus*, *Cyrtanthus montanus* (*C. montanus)*, *and Cyrtanthus odorus* (*C. odorus)* are found in specific vegetation types and soils.

Only two species of this taxon, namely *Cyrtanthus collinus* (*C. collinus)* and *Cyrtanthus ventricosus* (*C. ventricosus)* are well known, inhabiting the same soils and aspect in habitats on the continuous Cape Fold mountain ranges [328]. *Cyrtanthus collinus* is found on the coastal and inland mountains of the southern Cape and *C. ventricosus* extends from the Cape Peninsula into the Eastern Cape [328].

Most species of Clade C are found in the eastern lowlands and midlands of southern Africa, where they are concentrated in the subtropical biomes, Albany Thicket and Savanna [330,332]. This lineage constitutes *Cyrtanthus flammosus* (*C. flammosus)* and *Cyrtanthus spiralis* (*C. spiralis)*, which are narrowly widespread to the Albany Thicket Biome. Confined to the Savanna Biome are *Cyrtanthus eucallus* (*C. eucallus)* and *Cyrtanthus galpinii* (*C. galpinii)* in the Lowveld. Other species span the Albany Thicket and Savanna Biomes: the Eastern Cape *Cyrtanthus helictus* (*C. helictus)* and, extending northwards from the Albany region through South Africa, Zimbabwe, western Mozambique and East Africa into Sudan, is *Cyrtanthus sanguineus* (*C. sanguineus)* [328]. *Cyrtanthus obliquus*, adapted to nutrient-poor soils, occupies rocky habitats in east–west tending valleys. A summary of their geographic distribution is presented in Table 12.

### 5.3. Traditional Uses

*Cyrtanthus obliquus*, locally known as umathunga in South Africa, is used traditionally in the management of chronic coughs, headaches and scrofula [43,44]. *C. obliquus* root infusions are also employed in the management of stomach aches [333] while the crushed roots have been reported to find use in the management of leprosy [334]. *Cyrtanthus* species are also employed in the management of ailments associated with pregnancy, as well as cystitis, age-related dementia and leprosy [43,44]. Bulbs of *C. contractus* extracted in May and September is widely used locally in the management of mental illness, infections, inflammation, and cancer [335]. Infusions from species such as *C. breviflorus*, *C. contractus*, *C. mackenii*, *C. sanguineus*, *C. stenanthus* and *C. tuckii* are used by the Zulu in South Africa as protective sprinkling charms against storms and evil spirits [336]. Extracts of *C. breviflorus Harv.* are used as an anti-emetic agent and in the management of worm infestations such as tapeworm and roundworm. Extracts of *C. elatus* also finds use in the management of cough, headache and in labour induction [337].

### 5.4. Phytochemistry of Cyrtanthus

Species of *Cyrtanthus* have been identified as reservoirs for a host of chemical compounds. In a study performed by Mahlangeni et al., four homoisoflavanones, namely 5,7-dihydroxy-6-methoxy-3-(4′-methoxybenzyl)chroman-4-one, 5,7-dihydroxy-6-methoxy-3-(4′hydroxybenzyl)chroman-4-one and two 5,7-dihydroxy-6-methoxy-3-(4′-methoxybenzylidene)chroman-4-one, 5,7-dihydroxy-3-(4′hydroxybenzylidene)-chroman-4-one were isolated from the hexane, methanol and dichloromethane extracts of *Cyrtanthus obliquus* [338]. The bulbs of *C. obliquus* extracted with ethanol also revealed the presence of novel alkaloid obliquine, as well as 1α-hydroxygalanthamine, 3-epimacronine, narcissidine, tazettine and trisphaeridine [339].

The presence of lycorine, tazettine and 11-hydroxyvittatine in dried bulb ethanol extract of *Cyrtanthus mackenii* (Hook f.) has been demonstrated by Masi et al. [340]. Fresh bulb methanol extracts of *C. contractus* also contains a phenanthridone alkaloid called narciclasine [335]. Furthermore, two crinine alkaloids; haemanthamine and haemanthidine have been isolated from fresh bulb ethanol extracts of *C. elatus.* Further studies on the alcoholic extracts of the fresh bulbs also yielded the alkaloids zephyranthine, galanthamine and 1,2-O-diacetylzephyranthine [43,44]. Tazettine, maritidine, *O*-methylmaritidine, and papyramine are all phytochemicals that have been identified in fresh bulb methanol extracts of *C. falcatus* [337].

**Figure 6:**
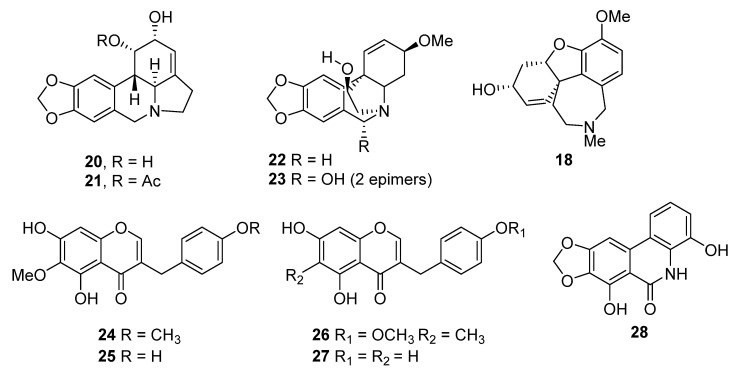
Chemical structures of selected compounds from *Crytanthus*. Zephyranthine (**20**), 1,2-*O*-diacetylzephyranthine (**21**), haemanthamine (**22**), haemanthadine (**23**), galanthamine (**18**), 5,7-dihydroxy-6-methoxy-3-(4ꞌ-methoxybenzyl)chroman-4-one (**24**), 5,7-dihydroxy-6-methoxy-3-(4ꞌ-methoxybenzylidene)chroman-4-one (**25**), 5,7-dihydroxy-6-methoxy-3-(4ꞌhydroxybenzyl)chroman-4-one (**26**), 5,7-dihydroxy-3-(4ꞌhydroxybenzylidene)-chroman-4-one (**27**) and naciprimine (**28**).

Chemical structure of compounds isolated from *Cyrtanthus* have been shown in Figure 6.

### 5.5. Pharmacological Activities

#### 5.5.1. Antioxidant Activity

5,7-dihydroxy-6-methoxy-3-(4′-methoxybenzyl)chroman-4-one and 5,7-dihydroxy-6-methoxy-3-(4′-hydroxybenzyl)chroman-4-one isolated from the fresh bulbs of *C. obliquus* have been shown to possess significant antioxidant activity with an *IC*_50_ of 371.54 and 288.40 µg/mL, respectively [338].

#### 5.5.2. Anti-Inflammatory Activity

The methanol extract of the bulbs of *C. contractus* has been investigated and shown to possess significant anti-inflammatory activity. The extract exhibited dose-dependent inhibition of E-selectin, a proinflammatory agent, when tested on endothelial cells. Further studies of the methanol extract on human umbilical vein endothelial cells revealed a concentration-dependent reduction in THP-1 adhesion via blockade of the expression of endothelial adhesion molecule ICAM-1. Narciclasine was identified as the main anti-inflammatory compound in the methanol extract of the bulbs of *C. contractus* [335].

The dichloromethane (DCM) extracts of *C. falcatus* (roots) and *C. mackenii* (leaves) were shown to interfere with the activity of cyclooxygenase 2 (COX-2) by at least 90%. DCM extract of *C. suaveolens* also blocked prostaglandin synthesis via antagonizing COX-2 activity by 81.6 %. Moderate inhibition (approximately 70%) of COX-2 activity was also observed with the methanol extracts of the roots and leaves of *C. falcatus* [341,342]. Selective inhibition of COX-2 by these extracts makes them suitable candidates for development for clinical use.

#### 5.5.3. Inhibition of Acetylcholinesterase

The phenanthridone alkaloid nacriprimine, isolated from the ethanolic bulb extract of *C. contractus* has been shown to possess mild acetylcholinesterase inhibition property with an *IC*_50_ of 78.9 µg/mL compared to the 40-fold more potent standard galanthamine with an *IC*_50_ of 1.9 µg/mL [11].

#### 5.5.4. Antimicrobial Activity

*Cyrtanthus* species and their isolated compounds have demonstrated noteworthy antimicrobial activity against a panel of microorganisms. *C. suaveolens* bulbs/roots and leaves isolated with DCM demonstrated broad-spectrum antimicrobial activity against *B. subtilis*, *E. coli*, *K. pneumoniae*, *M. luteus* and *S. aureus* with zones of inhibition ranging between 0.13–0.91 mm. DCM extracts of *C. falcatus* also inhibited the growth of *B. subtilis S. aureus* and *E. coli. C. mackenii* bulb/root extracts also inhibited the growth *M*. *luteus and S. aureus* [337].

Haemanthamine and haemanthidine isolated from the bulbs of *C. elatus* have been investigated for their activity against parasitic protozoans [43]. Haemanthamine showed activity against trophozoite stage of *Entamoeba histolytica* (*E. histolytica)* HK9 with an *IC*_50_ of 0.75 μg/mL and mild activity against *Plasmodium falciparum* (*P. falciparum)* NF54 with an *IC*_50_ of 0.67 μg/mL. The activity against *E. histolytica* was compared to ornidazole with an *IC*_50_ 0.28 μg/mL whiles the activity against *P. falciparum* was compared to chloroquine with an *IC*_50_ of 0.004 μg/mL and artemisinin with an *IC*_50_ of 0.002 μg/mL [43].

Haemanthidine also showed weak activity against *P. falciparum*, *T. brucei rhodesiense* STIB 900, and *T. cruzi* Tulahuen C4 with an *IC*_50_ of 0.70, 1.1 and 1.38 μg/mL, respectively. Melarsoprol with an *IC*_50_ of 0.002 μg/mL and benznidazole with an *IC*_50_ of 0.56 μg/mL were used as standards for *Trypanosoma brucei rhodesiense* STIB 900, and *Trypanosoma cruzi* Tulahuen C4, respectively [43].

#### 5.5.5. Cytotoxic Activity

Haemanthamine isolated from *C. elatus* was shown to possess cytotoxic activity which was mediated via the apoptotic pathway as depicted in rat hepatoma cell (5123tc). The *ED*_50_ was determined at 15 μM and this result was of particular interest due to its selectivity; haemanthamine demonstrated insignificant activity in normal human embryo kidney (293t) cells [337].

Alkaloids isolated from *C. obliquus* tested for cytotoxic activity against Chinese Hamster ovarian and human hepatoma (hepG2) cells showed no cytotoxic activity up to a concentration of 100 μg/mL [339].

Tazettine isolated from *C. falcatus* and other members of Amaryllidaceae has been reported to possess cytotoxic activity on colon cell line murine alveolar non-tumoral fibroblast [343,344]. Papyramine, also extracted from *C. falcatus* showed cytotoxic activity against murine alveolar non-tumoral fibroblast and human lymphoid neoplasm as well [343,344].

#### 5.5.6. Miscellaneous Pharmacological Activities

Roots of *C. falcatus* and *C. surveolens* extracted with DCM exhibited mutagenicity in *Salmonella* strain TA98 which was higher than that observed in the leaves of these plants. Mutagenicity was, however, not observed in the methanol extracts of these plants [337]. The mutagenicity of *C. suaveolens* has been attributed to the compound captan isolated from the bulbs/roots using DCM [344].

A summary of the traditional uses, phytochemicals and pharmacological activities of *Cyrtanthus* species have been highlighted in Table 13.

## 6. Conclusions

The discovery of new drugs in response to the growing burden of infectious and non-communicable diseases is of utmost necessity in this era. The genera *Tulbaghia*, *Allium*, *Crinum*, and *Cyrtanthus* of the Amaryllidaceae family have been well presented and shown to be a source of promising medicinal compounds with varying biological properties. Further research is therefore necessary to propel these compounds through clinical trials for possible usage in therapeutics. Although natural products have been attributed with high safety profiles, the presence of mutagenic compounds in crude extracts of these plants underscores the importance of pharmacological studies prior to their use in traditional medicine. These findings are relevant in light of augmenting the lean pipeline of drug discovery.

## Figures and Tables

**Table 1:** Geographical distribution and traditional uses of *Tulbaghia* species.

Plant Species	Geographical Distribution	Traditional Uses	References
*T. violacea*	Indigenous to the Eastern Cape, KwaZulu-Natal, Gauteng, Free State and Mpumalanga Provinces of South Africa.	The leaves and bulbs are used in the management of fever and colds, tuberculosis, asthma, and stomach problems. The leaves are eaten as vegetables and for the management of oesophageal cancer. It is also used as a snake repellent.	[8,50]
*T. alliacea*	Native to South Africa and grows mostly in the Eastern Cape and southern KwaZulu-Natal Provinces of South Africa.	Its bruised rhizome is used locally in bathwater to relieve fever, rheumatism, and paralysis, and in small doses as a laxative. *T. alliacea* is used for the management of stomach problems, asthma, and pulmonary tuberculosis. Its rhizome infusion is administered as an enema.	[8,51]
*T. simmleri*	Native to the South African Drakensberg mountains growing as isolated plants on rocky ledges.	Bulbs and leaves are used as a remedy for gastrointestinal ailments, enemas, high blood pressure, heart problems, chest complaints, high cholesterol, constipation, rheumatism, asthma, fever, pulmonary tuberculosis, earache, human immunodeficiency virus (HIV), paralysis, and cardiovascular diseases.	[50,52]
*T. acutiloba*	Found in the rainfall regions of southern Africa, occurring in the Eastern Cape, KwaZulu-Natal, Limpopo, Free State, Gauteng, North West, and Mpumalanga Provinces of South Africa, as well as in Lesotho, Swaziland and Botswana.	*T. acutiloba* leaves are used as a culinary herb and snake repellent. It is used to treat barrenness, flu, bad breath, and as an aphrodisiac. It is also cultivated to keep snakes away from the homestead.	[8]
*T. natalensis*	Although native to South Africa, but is now grown worldwide.	It is used as a culinary herb and snake repellent.	[53]
*T. cernua*	Commonly found in the Eastern Cape, Free State, Gauteng, KwaZulu-Natal, Limpopo, Mpumalanga, North West and Western Cape Provinces of South Africa.	It is used for ornamental purposes.	[8]
*T. leucantha*	Widely distributed in southern Africa including Botswana, Lesotho, South Africa, Swaziland, Zambia, and Zimbabwe.	Its rhizome is scraped clean and boiled in stews or roasted as a vegetable. Its leaves and stems are used as a culinary herb and protective charm.	[53]
*T. ludwigiana*	Commonly found in the Eastern Cape, KwaZulu-Natal, Northern Provinces of South Africa and in Swaziland.	It is traditionally used as a love charm.	[53]

**Table 2:** Published documents on the genus *Tulbaghia* per country.

Country	No. of Documents *
South Africa	99
United Kingdom	15
United States	12
Czech Republic	8
Italy	7
India	6
Germany	5
Australia	3
China	3
Belgium	2

* Data retrieved following query of the Scopus database (https://www.scopus.com/, accessed on 22 February 2022) using the keyword “*Tulbaghia*”. The search was carried out on 22 February 2022.

**Table 3:** Number of published studies per specific disease or pharmacological area.

Disease	No. of Published Studies #
Antimicrobial	26
Cancer	11
Antioxidant	13
Diabetes	2
Cardiovascular	12
Antithrombogenic	2
Miscellaneous	17

# Studies considered are those published from 1997 to 2022. A number of these, published before 2013, have been succinctly discussed by Aremu and Van Staden [8].

**Table 4:** Antimicrobial activity of *Tulbaghia* species.

Plant Species	Extraction Solvent	Plant Part Used	Biological Activity	References
*T. violacea*	Dichloromethane	Bulbs	MIC ranging from 20 to 300 µg/mL against *Bacillus subtilis*, methicillin-resistant *S. aureus*, *S. epidermidis*, *E. coli*, *K. pneumoniae*, *P. aeruginosa*, *C. albicans* and *C. parapsilosis*.	[67]
*T. violacea*	Hexane and ethanol	Flowers and callus cultures	Moderate to strong broad-antimicrobial (*E. coli*, *P. aeruginosa*, *S. aureus*, *Aspergillus niger* and *C. albicans*) activity observed by zone of inhibition in the agar well disc diffusion method.	[68]
*T. violacea*	Water	Bulbs	Significant reduction in *A. flavus* β-glucan and chitin synthesis corresponding to a dose-dependent inhibition of the enzymes β-glucan and chitin synthase, respectively.This results in inhibition of ergosterol production in the fungus.	[70,71]
*T. violacea*	Acetone	Bulbs	Varied light intensities, pH and watering frequencies substantially impacted both growth and potency of plant extracts against the fungi *F. oxysporum*.	[73,74]
*T. violacea*	Water	Roots, bulbs, leaves and flowers	Significantly compromised population densities of the nematode *M. incognita* race 2 on tomato roots and in the soil.	[75]
*T. violacea*	Dichloromethane	Bulbs	Antiparasitic activity against *T. brucei* (*IC*_50_ = 2.83 µg/mL) and *L. tarentolae* (*IC*_50_ = 6.29 µg/mL).	[67]

**Table 5:** Anticancer activity of *Tulbaghia*.

Plant Species	Extraction Solvent	Plant Part Used	Biological Activity	References
*T. violacea*	Methanol	Leaves and roots	Marked time- and dose-dependent cytotoxic effect on cancer cell lines. Induced apoptosis using p53-independent pathway.	[78]
*T. violacea*	Methanol, butanol, and hexane	Leaves	Methanol extract was prolific against multiple cell lines. Hela and ME-180 cell lines treated with methanol and hexane extracts showed an increase in caspase 3/7 activity. Both methanol and hexane extracts induced a 10-fold increase in expression of p53 gene in Hela cells.	[79]
*T. violacea*	Methanol:water:formic acid (80:20:0.1, *v/v/v*)	Flowers	Demonstrated activity against ovarian tumor cells.	[80]
*T. violacea*	Water and methanol	Leaves	Water-soluble extract emerged as the most cytotoxic (*IC*_50_ = 314 µg/mL), compared to the methanol extract (*IC*_50_ = 780 µg/mL), against the MDA-MB-231 triple-negative breast cancer cell line. Water-soluble extract prevented cell migration completely for 13 h at 300 µg/mL.	[82]
*T. violacea*	Hexane and ethanol	Flowers and callus cultures	Extracts showed marked cytotoxicity (60–74% growth inhibition at 250 µg/mL) against three different cell lines (Hep G2, PC-3 and MCF-7).	[68]
*T. violacea*	Acetone and water	Leaves	Anticancer activity against oral cancer with an *IC*_50_ (acetone extract) of 0.2 mg/mL; *IC*_50_ (water extract) of 1 mg/mL.	[84]
*T. violacea*	Methanol:water (1:1)	Whole plants	Two pro-apoptotic glucopyranosides d-fructofuranosyl-β (2→6)-methyl-α-d-glucopyranoside and β-d-fructofuranosyl-(2→6)-α-d-glucopyranoside isolated and identified as active anticancer agents in the plant.	[85]
*T. violacea*	Water	Whole plants	MoA of the three compounds, namely methyl-α-d-glucopyranoside, d-fructofuranosyl-β (2→6)-methyl-α-d-glucopyranoside and β-d-fructofuranosyl-(2→6)-α-d-glucopyranoside isolated from the water extract, deciphered to be through induction of apoptosis by targeting the mitochondrial (intrinsic) pathway	[86]

**Table 6:** Antioxidant activity of *Tulbaghia* species.

Plant Species	Extraction Solvent	Plant Part Used	Biological Activity	References
*T. violacea*	Water	Leaves	Dose-dependent antioxidant activity measured using the DPPH (Log *IC*_50_ = 0.49 mg/mL) and ABTS (Log *IC*_50_ = 0.24 mg/mL) assays	[92]
*T. violacea*	Methanol/water/formic acid (80:20:0.1, *v/v/v*)	Flowers	Marked antioxidant activity was observed using 3 different types of assays, namely DPPH, FRAP and TREC	[80]
*T. acutiloba*	Hydro-methanolic extracts	Roots, rhizomes, leaves and flowers	Dose-dependent antioxidant activity observed with the rhizome extract emerging as the most active plant part (*IC*_50_ DPPH = 0.202 mg/mL and peak scavenging activity of 95)	[91]
*T. violacea*	Hexane and ethanol	Flowers and callus cultures	Dose-dependent antioxidant activity with *IC*_50_ ranging from 1.933 to 7.350 mg/mL in the DPPH assay	[68]
*T. violacea*	Acetone	Leaves	*IC*_50_ DPPH = 0.08 mg/mL; *IC*_50_ ABTS = 0.03 mg/mL	[84]
*T. acutiloba*	Acetone	Leaves	*IC*_50_ DPPH = 0.16 mg/mL; *IC*_50_ ABTS = 0.07 mg/mL	[84]
*T. alliacea*	Acetone	Leaves	*IC*_50_ DPPH = 0.06 mg/mL; *IC*_50_ ABTS = 0.06 mg/mL	[84]
*T. cernua*	Acetone	Leaves	*IC*_50_ DPPH = 0.21 mg/mL; *IC*_50_ ABTS = 2.34 mg/mL	[84]
*T. leucantha*	Acetone	Leaves	*IC*_50_ DPPH = 0.39 mg/mL; *IC*_50_ ABTS = 0.03 mg/mL	[84]
*T. ludwigiana*	Acetone	Leaves	*IC*_50_ DPPH = 0.26 mg/mL; *IC*_50_ ABTS = 0.09 mg/mL	[84]
*T. natalensis*	Acetone	Leaves	*IC*_50_ DPPH = 2.70 mg/mL; *IC*_50_ ABTS = 0.04 mg/mL	[84]

**Table 7:** Antidiabetic, anticardiovascular and antithrombogenic activity of *Tulbaghia* species.

Plant Species	Extraction Solvent	Plant Part Used	Biological Activity	References
Diabetes				
*T. violacea*	Methanol	Rhizome	Attenuated diabetes associated physiological complications in streptozotocin-induced diabetic rats.	[96]
*T. violacea*	Methanol	Rhizome	Noticeably reduced blood glucose and serum lipid (TG, TC, and VLDL) levels while raising plasma insulin in a streptozotocin-induced diabetic rat model.	[97]
Cardiovascular				
*T. violacea*	Methanol	Leaves	Markedly reduced systolic BP, diastolic BP, mean arterial pressure and the heart rate in both age-induced and spontaneous hypertensive rats.	[98]
*T. violacea*	Methanol	Rhizome	50 mg/kg significantly improved kidney function in vivo.	[99]
*T. acutiloba*	Hydro-methanolic extracts	Roots, rhizomes, leaves and flowers	All extracts inhibited the Angiotensin-1-Converting Enzyme in vitro (> 50 % inhibition at a concentration range of 125–1000 μg/mL). Extracts of leaves demonstrated activity comparable to that of the control drug ramipril.	[91]
Antithrombogenic				
*T. violacea*	Water	Leaves	Noticeable inhibition of platelet adhesion by a novel scaffold consisting of polycaprolactone incorporated with 10 % (*w*/*w*) plant extracts.	[90]
*T. violacea*	Water	Leaves	Marked inhibition of platelet adhesion (70% inhibition at 0.1 mg/mL within 15 min post-exposure).	[92]

**Table 8:** Miscellaneous biological properties of extracts of *Tulbaghia* species.

Plant Species	Extraction Solvent	Plant Part Used	Biological Activity	References
*T. violacea*	Methanol/water/formic acid (80:20:0.1, *v*/*v*/*v*)	Flowers	Reduced 1-42 β-amyloid peptide formation and arrested oxidative stress in vivo.	[80]
*T. violacea*	Methanol	Leaves	Demonstrated in vivo anticonvulsant activity by attenuating tonic convulsions induced by either pentylenetetrazole, bicuculline, picrotoxin, strychnine or NMDLA.	[102]
*T. violacea*	Acetone	Mixture of leaves and bulbs	Marked tick repellence properties of fungus-exposed plants at low treatment concentrations (5 % *w/v* and 10 % *w/v*).	[59]
*T. violacea*	Water	Leaves, stems, and roots	Induced conspicuous genotoxicity effects at high test concentrations (250, 500 and 1000 µg/mL) in the *A. cepa* assay.	[103]
*T. violacea*	Water and ethanol	Leaves, stems, and roots	Broad murine macrophage antiproliferative and cytotoxicity activity influenced by both extract test concentrations, type of solvent and plant part used.	[104]

**Table 9:** Traditional medicinal uses of *Allium* species.

Plant Species	Mode of Preparation	Traditional Medicinal Uses	Reference
*A. cepa*	Raw, juice of bulb or rhizome, paste, decoctions, cataplasm, maceration, infusion	Alopecia, antilithic (stone disease), anti-obesity, blood purifying, bronchitis, constipation, cardiovascular disease, cough, diabetes, eye diseases, erectile dysfunction, fever, hearing loss, headaches, hemorrhoids, epilepsy, oligomenorrhea, jaundice, lower gastrointestinal bleeding, prostate cancer, rheumatism, rubefacient, sinusitis, stomach pains, snake bites, skin diseases, teeth disorders, reduce flatulence, wound healing	[133,134,135]
*A. sativum*	Extracts of leaves or bulb	Antiseptic, anthelmintic, antithrombotic, antilipidemic, aphrodisiac, anti-greying of hair, bronchitis, carminative, cough, colic, cancers (gastric, prostate, colorectal adenomatous polyps, squamous cell carcinoma), diabetes, diaphoretic, dysentery, eczema, facial paralysis, fever, flatulence, galactagogue, high blood pressure, intestinal worms, liver disorders, rheumatism, scabies, tetanus, stomach pains, tuberculosis	[135,136,137,138,139]
*A. umbilicatum*	Raw or cooked bulb, leaves, flowers	Non-specific reduction in blood cholesterol levels, tonify digestive and circulatory systems	[116]
*A. victoralis*	Fresh, pickled, boiled and salted flowers, leaves and roots	Appetizer, amenorrhea, pediatric otitis, bronchitis, diarrhea, dropsy, expectorant, hypofunction of stomach, inflammatory eye diseases, meteorism, gastroenteritis, heart diseases (atherosclerosis), rheumatism	[140]
*A. ascalonicum*/*A. cepa var aggregatum*	Bulb and leaves	Allergies, appetizer, cold, cancers, fever, obesity, rheumatoid arthritis, soothes nerves, diabetes, post-menopausal syndrome	[141,142,143,144,145]
*A. chinense*	Flower, leaves, roots, seedpods	Angina pectoris, astringent, bronchitis, carminative, chest pains, diarrhea, expectorant, pleurisy, tenesmus in cases of dysentery, reducing cholesterol, tonic to the digestive and circulatory systems	[146]
*A. tuberosum*	Raw or cooked leaves, roots, oils from seed	Asthma, abdominal pain, carminative, cuts and wounds, diabetes, diarrhea, kidney and bladder weakness, nocturnal emission, urinary incontinence, spermatorrhea, stomachic	[147]
*A. griffithianum*	Leaves and bulb	Carminative, colic indigestion, dyspepsia, diabetes control	
*A. oreoprasum*	Leaves and bulb	Cough and cold, diabetes control, diarrhea, dysentery, fever, gastritis, oedema, headache, jaundice, stomachache, rheumatism, numbness of limbs	[124]

**Table 10:** Bioactive compounds isolated from *Allium* species.

Plant Species	Plant Part	Country	Isolated Compounds	Bioactivity	References
*A. ursinum* L.	Leaves, underground parts, fresh flowers	Poland Bulgaria	1,2-di-*O*-α-linolenoyl-3-*O*-β-d-galactopyranosyl-sn-glycerol; β-sitosterol3-*O*-β-d-glucopyranoside; kaempferol 3-*O*-β-glucopyranoside and kaempferol 3-*O*-β-neohesperidoside. (-S-)-spirost-5-en-3β-ol tetrasaccharide, (25*R*)-spirost-5,25(27)-dien-3 β-ol tetrasaccharide, 3-hydroxypregna-5,16-dien-20-one glycoside. Thymidine, adenosine, astragalin (kaempferol-3-*O*-β-d-glucopyranoside, kaempferol-3-*O*-β-d-glucopyranosyl-7-*O*-β-d-glucopyranoside, kaempferol-3-*O*-β-d-neohesperoside, and kaempferol-3-*O*-β-d-neohesperoside-7-*O*-β-d glucopyranoside.	Anti-ADP-aggregation activity in human blood platelets. Inhibition of human platelet aggregation. Cytotoxic activity against murine melanoma B16 and sarcoma XC.	[148,149,150,151]
*A. mongolicum*	Aerial parts	China	Mongoflavonoids A_1_, A_2_, A_3_, A_4_, B_1_, B_2_ and monogophenosides A_1_, A_2_, A_3_, B.	Increase in the height of mouse small intestine.	[152]
*A. cepa.* *A. cepa L.*	Pigmented scales of red onion, bulbs, red onion skin waste	Naples	Quercetin. 3-*O*-(3″-*O*-β-glucopyranosyl-6″-*O*-malonyl-β-glucopyranoside)-4-*O*-β-glucopyranoside, cyanidin 3,4′-di-*O*-β-glucopyranoside, cyanidin-4′-*O*-β-glucoside, peonidin 3-*O*-(6 ″-*O*-malonyl-β-glucopyranoside). 5-hydroxy-3-methyl-4-propylsulfanyl-5H-furan-2-one, (hydroxymethyl) furfural, acetovanillone, methyl 4-hydroxyl cinnamate and ferulic acid methyl ester. 3-*O*-β-glucopyranoside and 3-*O*-(6″-*O*-malonyl-β-glucopyranoside) of 5-carboxypyranocyanidin. Ceposide A, ceposide B and ceposide C. Spiraeoside (4′-*O*-glucoside of quercetin). Onionin A_1_, onionin A_2_, onionin A_3_, onionin B_1_ and B_2_. Onionin A_1_ (3,4-dimethyl-5-(1E-propenyl)-tetrahydrothiophen-2-sulfoxide-*S*-oxide). Cyanidin 3-glucoside (Cy 3-Glc), 3-malonylglucoside (Cy3-MaGlc), cyanidin 3-laminaribioside (Cy 3-Lam) and 3-malonyllaminaribioside (Cy 3-MaLam).	Anti-inflammatory and immunomodulatory effect. Induction of quinone reductase. Antifungal activity. Radical scavenging, anti-inflammatory, inhibition of the expression of B-cell lymphoma 2. Suppression of tumor progression in mouse ovarian cancer (Onionin A_1_). Suppression of tumor-cell proliferation through the inhibition of polarization of M_2_ activated macrophages.	[153,154,155,156,157,158,159,160,161]
*A. sativum.* *A. sativum L. var. voghiera.* *A. sativum L.*	Root, protobulb, leaf sheath and blade, bulbs, tuber	Italy	Nerolidol, α-pinene, terpinolene. Voghieroside A1/A2, voghieroside B1/B2, voghieroside C1/C2, voghieroside D1/D2 and voghieroside E1/E2. Adenosine and guanosine.	Antifungal activity against *Sclerotium cepivorum*. Antimicrobial activity. Strong inhibitory effect on human platelet aggregation generated by 2 μM ADP in both primary and secondary waves (adenosine).	[162,163,164]
*A. schoenoprasum*	Whole plant, pale-purple flowers		(20S, 25S)-spirost-5-en-3β, 12β,21-triol 3-*O*-α-L-rhamnopyranosyl-(1→2)-β-d-glucopyranoside, (20*S*, 25*S*)-spirost-5-en-3β, 11α,21-triol 3-*O*-α-L-rhamnopyranosyl-(1→2)-β-d-glucopyranoside, laxogenin 3-*O*-α-L-rhamnopyranosyl--(1→2)-[β-d-glucopyranosyl-(1→4)]-[β-d-glucopyranoside, (25*R*)-5α-spirostan-3β, 11α-diol 3-*O*-β-d-glucopyranosyl-(1→4)]-β-d-galactopyranoside. (cyanidin 3-*O*-β-glucosideAII) (kaempferol 3-*O*-(2-*O*-β-glucosylFIII-β-glucosideFII)-7-*O*-β-glucosiduronic acid FIV) malonate AIII (AII-6→AIII-1, FIV-2→AIII-3), 1, (cyanidin 3-*O*-(3-*O*-acetyl-β-glucosideAII) (kaempferol 3-*O*-(2-*O*-β-glucosylFIII-β-glucosideFII)-7-*O*-β-glucosiduronic acid FIV) malonate AIII (AII-6→AIII-1, FIV-2→AIII-3), 2, and 7-*O*-(methyl-*O*-β-glucosiduronateFIV).	Cytotoxicity against HCT 116 and HT-29 human colon cancer lines.	[165,166]
*A. minutiflorum Regel*	Bulbs		Minutoside A, minutoside B, Minutoside C, alliogenin, neoagigenin	Antifungal activity.	[167]
*A. neapolitanum*	Extracts		3-*O*-{[2-*O*-α-1-rhamnopyrnosyl-4-*O*-β-d-glucopyranosyl]-β-d-glucopyranoside}, isorhamnetin; 3-*O*-{[2-*O*-α-1-rhamnopyrnosyl-6-*O*-β-d-glucopyranosyl]-β-d-glucopyranoside}, isorhamnetin; 3-*O*-{[2-*O*-α-1-rhamnopyranosyl-4-*O*-β-d-glucopyranosyl]-β-d-glucopyranoside}-7-*O*-β-d-glucopyranoside and isorhamnetin; 3-*O*-{[2-*O*-α-1-rhamnopyranosyl-6-*O*-β-d-gentiobiosyl]-β-d-glucopyranoside}.	Antiplatelet aggregation activity.	[168]
*A. tripedale*	Bulbs, leaves	Iran	6,7-dimethoxy-*N*-trans-caffeoyltyramine; *N*-trans-feruloyltyramine. (+)-*S*-(1-butenyl)-*L*-cysteine sulfoxide (homoisoalliin), *S*-(1-butenyl)-*L*-cysteine (desoxyhomoisoalliin).	NR.	[167,168]
*A. porrum* L.	Bulbs		Kaempferol 3-*O*-[2-*O*-(trans-3-methoxy-4-hydroxycinnamoyl)-β-d-galactopyranosyl]-(1→4)-*O*-β-d-glucopyranoside; Kaempferol 3-*O*-[2-*O*-(trans-3-methoxy-4-hydroxycinnamoyl)-β-d-glucopyranosyl]-(1→6)-*O*-β-d-glucopyranoside. (25*R*)-5 α-spirostan-3 β, 6 β-diol 3-*O*-[*O*-β-d-glucopyranosyl-(1→2)-*O*-[β-d-xylopyranosyl-(1→3)]-*O*-β-d-glucopyranosyl-(1→4)-β-d-galactopyranoside}; (25*R*)-5 α-spirostan-3 β, 6 β-diol 3-*O*-{*O*-β-d-glucopyranosyl-(1→3*)-O-*β-d-glucopyranosyl-(1→2)-*O*-[β-d-xylopyranosyl-(1→3)]-*O*-β-d-galactopyranosyl-(1→4)-β-d-galactopyranoside}	Antiplatelet aggregation activity. Antifungal activity.	[169,170,171]
*A. chinense.* *A. chinense* G. Don	Bulbs		Chinenoside II and chinenoside III. (25 *R*,*S*)-5 α-Spirostan-3β-ol tetrasaccharide, (25*R*)-3 β-hydroxy-5 α-spirostan-6-one di- and tri-saccharides. Xiebai-saponin I (laxogenin 3-*O*-β-xylopyranosyl (1→4)-[α-arabinopyranosyl (1→6)-β-glucopyranoside), laxogenin 3-*O*-α-arabinopyranosyl (1→6)-β-glucopyranoside, laxogenin, isoliquiritigenin, isoliquiritigenin-4-*O*-glucoside, and β-sitosterol glucoside.	Inhibition of cAMP phosphodiesterase. Antitumor-promoting activity (laxogenin).	[172,173,174,175]
*A. macrostemon.* *A. macrostemon* Bunge	Bulbs, leaves	Japan	Macrostemonoside G (26-*O*-β-d-glucopyranosyl-22-hydroxy-5-β-furost-25(27)-ene-3 β,12 β,26 triol 3-*O*-β-d-glucopyranosyl(1-->2)-β-d-galactopyranoside) and I (26-*O*-β-d-glucopyranosyl-22-hydroxy-5 β-furost-25(27)-ene-12-one-3 β,26-diol 3-*O*-β-d-glucopyranosyl(1→2)-β-d-galactopyranoside). tigogenin-3-*O*-β-d-glucopyranosyl(1-->2) [β-d-glucopyranosyl(1→3)1]-β-d-glucopyranosyl(1→4)-β-d-galactopyranoside (1) and tigogenin-3-*O*-β-d-glucopyranosyl(1→2)[β-d-glucopyranosyl (1→3)(6-*O*-acetyl-β-d-glucopyranosyl)] (1→4)-β-d-galactopyranoside (2). Macrostemonoside E-(25*R*)-26-*O*-β-d-glucopyranosyl-5 α-furost-20(22)-ene-3 β,26-diol-3-*O*-β-d-glucopyranosyl (1→2) [β-d-glucopyranosyl (1→3)]-β-d-glucopyranosyl (1→4)-β-d-galactopyranoside; Macrostemonoside F(II)-(25*R*)-26-*O*-β-d-glucopyranosyl-5 β-furost-20(22)-ene-3 β,26-diol-3-*O*-β-d-glucopyranosyl (1→2)-β-d-galactoside. Allimacronoid A (1-*O*-(*E*)-feruloyl-β-d-glucopyranosyl (1-2)-[β-d-glucopyranosyl (1-6)]-β-d-glucopyranose), Allimacronoid B (1-*O*-(*E*)-feruloyl-{β-d-glucopyranosyl (1-4)-[β-d-glucopyranosyl (1-2)]}-[β-d-glucopyranosyl (1-6)]-β-d-glucopyranose) and Allimacronoid Cn1-*O*-(*E*)-feruloyl-{β-d-glucopyranosyl (1-6)-[β-d-glucopyranosyl (1-2)]}-[β-d-glucopyranosyl (1-6)]-β-d-glucopyranose.	In vitro inhibition of ADP-induced human platelet aggregation (macrostemonoside G). Inhibitory activity against rabbit platelet aggregation induced by ADP (1).	[176,177,178,179]
*A. schubertii*	Bulbs		(25*R* and *S*)-5 α-spirostan-2 α,3 β,6 β-triol 3-*O*-β-d-glucopyranosyl-(1→2)-*O*-[4-*O*-benzoyl-β-d-xylopyranosyl-(1→3)]-*O*-β-d-glucopyranosyl-(1→4)-β-d-galactopyranoside, (25*R* and *S*)-5 α-spirostan-2α,3β,6 β-triol 3-*O*-β-d-glucopyranosyl-(1→2)-*O*-[3-*O*-benzoyl-β-d-xylopyranosyl-(1→3)]-*O*-β-d-glucopyranosyl-(1→4)-β-d-galactopyranoside, (25*R* and *S*)-5 α-spirostan-2α,3β,6 β-triol 3-*O*-β-d-glucopyranosyl-(1→2)-*O*-[4-*O*-(3*S*)-3-hydroxy-3-methylglutaroyl-β-d-xylopyranosyl-(1→3)]-*O*-β-d-glucopyranosyl-(1→4)-β-d-galactopyranoside and 26-*O*-β-d-glucopyranosyl-(25*R* and *S*)-5 α-furostan-2α,3β,6β,22 zeta,26-pentol 3-*O*-β-d-glucopyranosyl-(1→2)-*O*-[β-d-xylopyranosyl-(1→3)]-*O*-β-d-glucopyranosyl-(1→4)-β-d-galactopyranoside.	NR.	[180]
*A. tuberosum*	Seeds	Shanghai	(2α, 3β, 5α, 25*S*)-2,3,27-trihydroxyspirostane 3-*O*-α-*L*-rhamnopyranoyl-(1→2)-*O*-[α-*L*-rhamnopyranosyl-(1→4)]-β-d-glucopyranoside. Tuberoside J-(25*R*)-5 α-spirostan-2α,3 β,27-triol 3-*O*-α-*L*-rhamnopyranosyl-(1-->2)-β-d-glucopyranoside; Tuberoside K-(25*R*)-5α-spirostan-2α,3β 27-triol 3-*O*-α-*L*-rhamnopyranosyl-(1→2)-[α-*L*-rhamnopyranosyl-(1→4)]-β-d-glucopyranoside; and Tuberoside L-27-*O*-β-d-glucopyranosyl-(25*R*)-5α-spirostan-2α,3 β,27-triol 3-*O*-α-d-rhamnopyranosyl-(1-->2)-[α-*L*-rhamnopyranosyl-(1→4)]-β-d-glucopyranoside. Tuberoside M-(25*S*)-5β-spirostane-β,3 β-diol 3-*O*-α-L-rhamnopyranosyl-(1→4)-β-d-glucopyranoside. Tuber-ceramide (*N*-(2′,3′-dihydroxy-tetracosenoyl)-2-amino-1,3,4-trihydroxy octadecane), and Cerebroside (*N*-(2′,3′-dihydrox-tetra-cosenoyl)-2-amino-1,3,4-trihydroxy octadecane).	Tuberoside M inhibits the proliferation of the human promyelocytic leukemia cell line (HL-60)	[181,182,183]
*A.albopilosum* and *A. ostrowskianum*	Bulbs		(25 *R* and *S*)-5 α-spirostane-2α, 3 β,6 β-triol 3-*O*-(*O*-β-d-glucopyranosyl-(1→2)-*O*-[3-*O*-acetyl-β-d-xylopyranosyl-(1→3)]-*O*-β-d-glucopyranosyl-(1→4)-β-d-galactopyranoside), (25*R*)-2-*O*-[(*S*)-3-hydroxy-3-methylglutaroyl]-5 α-spirostane-2α, 3β, 6β-triol 3-*O*-(*O*-β-d-glucopyranosyl-(1→2)-*O*-[β-d-xylopyranosyl-(1— >3)]-*O*-β-d-glucopyranosyl-(1→4)-β-d-galactopyranoside), (22*S*)-cholest-5-ene-1β, β,16 β,22-tetraol 1-*O*-α-*L*-rhamnopyranoside 16-*O*-(*O*-α-*L*-rhamnopyranosyl-(1→3)-β-d-glucopyranoside), 1β, 3β, 16β-trihydroxycholest-5-en-22-one 1-*O*-aα-L-rhamnopyranoside 16-*O*-(*O*-α-*L*-rhamnopyranosyl-(1→3)-β-d-glucopyranoside), 1β,3β,16 bβ-trihydroxy-5 α-cholestan-22-one 1-*O*-α-*L*-rhamnopyranoside 16-*O*-(*O*-α-*L*-rhamnopyranosyl-(1→3)-β-d-glucopyranoside) and (22*S*)-cholest-5-ene-1β,3β, 16β,22-tetraol 16-*O*-(*O*-β-d-glucopyranosyl-(1→3)-β-d-glucopyranoside).	NR.	[184]
*A.fistulosum.* *A.fistulosum* L.	Whole plant, leaves, seeds	Iran	Fistulomidate A ((1*Z*,2*E*)-Methyl3-(3,4-dimethoxyphenyl)-*N*-(4-hydroxyphenethyl) acrilimidate) and Fistulomidate B ((1Z,2E)-Methyl3-(3,4-dihydroxyphenyl)-*N*-(4-hydroxyphenethyl)acrilimidat). Onionin A_1_, onionin A_2_, and onionin A_3_. Glycerol mono-(E)-8,11,12-trihydroxy-9-octadecenoate, tianshic acid, 4-(2-formyl-5-hydroxymethylpyrrol-1-yl) butyric acid, p-hydroxybenzoic acid, vanillic acid, and daucosterol.	Antibacterial and cytotoxic activity. Suppression of tumor progression in mouse ovarian cancer (onionin A_1_). Inhibition of the growth of *Phytophtohora capsici* on V8 media (glycerol mono-(E)-8,11,12-trihydroxy-9-octadecenoate and V).	[159,185,186]
*A.carolinianum* DC	Bulb	Mongolia	Cinnamoylphenethylamine derivative	Weak cytotoxic activity	[187]
*A.ampeloprasum var. porrum* (*Leek)*	Plant parts		A-β- d -glucopyranoside	Anticancer activity against MCF-7 human breast cancer cell.	[187]
*A.ascalonicum* L.		China	Ascalonicoside C-(25*R*)-26-*O*-β-d-glucopyranosyl-22-hydroxy-5α-furost-2-one-3β,5,6β, 26-tetraol-3-*O*-α-*L*-rhamnopyranosyl-(1→2)-β-d-glucopyranoside. Ascalonicoside d-(25*R*)-26-*O*-β-d-glucopyranosyl-22-methoxy-5α-furost-2-one-3β,5,6β, 26-tetraol-3-*O*-α-L-rhamnopyranosyl-(1→2)-β-d-glucopyranoside. (25*R*)-26-*O*-β-d-glucopyranosyl-22-hydroxy-5-ene-furostan-3β,26-diol-3-*O*-α-L-rhamnopyranosyl-(1→4)-α-L-rhamnopyranosyl-(1→4)-[ α-L-rhamnopyranosyl-(1→2)]-β-d-glucopyranoside. 25*R*)-26-*O*-β-d-glucopyranosyl-22-hydroxy-5-ene-furostan-3β, 26-diol-3-*O*-α-L-rhamnopyranosyl-(1→2)-[α-L-arabinofuranosyl-(1→4)]-β-d-glucopyranoside.	NR.	[188,189]
*A.* *siculum*	Bulbs	Zwanenburg, The Netherlands	(*Z*)-Butanethial *S*-oxide, (*R*(S),*R*(C),E)-*S*-(1-butenyl)cysteine *S*-oxide (homoisoalliin).	NR.	[190]
*A.* *chrysanthum*	Barks	Guangzhou, China	Chrysanthumones A (6″,6″-dimethyl-4″,5″-dihydropyrano [2″,3″: 8,7]-6″′,6″′-dimethyl-prenyl-4″′,5″′-dihydropyrano [2″′,3″′:2′,3′]apigenin) and B ((*E*)-5,7-dihydroxy-2-(4-hydroxyphenyl)-8-(3-methylbut-1-enyl)-4H-chromen-4-one).	NR.	[191]
*A.* L. *melanocrommyum* section *Megaloprason.*	Bulbs	Central Asia	*L*-(+)*-S-*(2-pyridyl)-cysteine sulfoxide.	NR.	[192]
*A.ampeloprasum* L.	Bulbs	United States of America	Ampeloside Bs_1_ (apigenin 3-*O*-β-glucopyranosyl (1 → 3)-β-glucopyranosyl (1 → 4)-β-galactopyranoside), ampelosides Bf_1_ ((25*R*)-26-*O*-β-glucopyranosyl-22-hydroxy-5α-furostane-2α,3β,6β,26-tetraol-3-*O*-β-glucopyranosyl(1 → 3)-β-glucopyranosyl-(1 → 4)-β-galactopyranoside) and Bf_2_ ((25*R*)-26-*O*-β-glucopyranosyl-22-hydroxy-5α-furostane-2α,3β,6β,26-tetraol-3-*O*-β-glucopyranosyl(1 → 4)-β-galactopyranoside).	Weak antifungal activity by ampeloside Bs_1_.	[193]
*A.bakeri* Reg.	Tuber		Adenosine, guanosine, and tryptophan, β-sitosterol β-d-glucoside.	Strong inhibitory effect on human platelet aggregation generated by 2 μM ADP in both primary and secondary waves (adenosine).	[162]
*A.* *victorialis var. platyphyllum*	Aerial parts, bulbs	Korea	Gitogenin 3-*O*-lycotetroside, astragalin and kaempferol 3, 4′-di-*O*-β-d-glucoside.	Cytotoxic activity.	[194]
*A.nutans* L.	Underground plant parts		Deltoside, nolinofuroside D, 25*R* Δ(5)-spirostan 3β-ol-3-*O*-α-L-rhamnopyranosyl(1-->2)-[β-d-glucopyranosyl(1→4)]-*O*-β-d-galactopyranoside and 25*R* Δ(5)-spirostan 1 β, 3β-diol 1-*O*-β-d-galactopyranoside.	NR.	[195]
*A.* *giganteum*	Bulbs	Japan	3-*O*-acetyl-(24*S*,25*S*)-5α-spirostane-2α,3β,5α,6β,24-pentol 2-*O*-β-d-glucopyranoside.	Inhibition of cAMP phosphodiesterase activity.	[196]
*A.hookeri* Thwaites	Rhizomes	China	Di-2-propenyl trisulfide, diallyl disulfide, and dipropyl trisulfide.	Antimicrobial activity against *Aspergillus fumigatus* and *C. albicans*.	[197]

NR: not reported.

**Table 11:** Bioactive compounds isolated from *Crinum* species.

Plant Species	Plant Part	Country	Isolated Compounds	Bioactivity	References
*C.* x *amabile* *C.* x *amabile* Donn ex Ker Gawl	Bulbs Stems, roots	Ecuador Brazil Thailand	Haemanthamine/crimine-type alkaloid. Lycorine-type alkaloid Galanthamine-type alkaloid. Augustine *N*-oxide, buphanisine *N*-oxide. Amabiloid A.	Anticholinesterase (anti-AChE) and antibutyrylcholinesterase (anti-BuChE) activity.	[249,250,251]
*C. defixum* Ker-Gawl	Bulbs	India	Hydrazide derivative. (*E*)-*N*-[(*E*)-2-butenoyl]-2-butenoylhydrazide.	Anti-genotoxic activity.	[252]
*C. moorei*	Bulblets		Cherylline, crinamidine, crinine, epibuphanisine, lycorine, powelline, undulatine, 1-epideacetylbowdensine, 3-*O*-acetylhamayne. 3-[4′-(8′-aminoethyl) phenoxy] bulbispermine, mooreine.	NR.	[253]
*C. biflorum*	Bulbs	Senegal	5,6,7-trimethoxy-3-(4 hydroxybenzyl) chroman-4-one, 3-hydroxy-5,6,7-trimethoxy-3-(4-hydroxybenzyl) chroman-4-one, 3-hydroxy-5,6,7-trimethoxy-3-(4-methoxybenzyl) chroman-4-one, 5,6,7-trimethoxy-3-(4-methoxybenzyl) chroman-4-one, (*E*)-*N*-(4-hydroxyphenethyl)-3-(4-hydroxyphenyl) acrylamide.	Anticancer, anti-AChE, anti-glucosidase activity.	[254,255]
*C. asiaticum* *C. asiaticum* var. *sinicum* *C. asiaticum* L. *C. asiaticum* var. japonicum. *C. asiaticum* L. var*. sinicum*.	Seeds, rhizome, fruits Bulbs, stems, leaves	Beijing, China, Hainan Province, Japan, Island of Jeju in Korea	Flavonoids Isopowellaminone. (2*R*,3*S*)-7-methoxyflavan-3-ol (1:), (2*R*,3*S*)-7-hydroxy-flavan-3-ol (2:), (2*R*,3*S*)-2 ′-hydroxy-7-methoxy-flavan-3-ol (3:). Norgalanthamine. Crinamine CAL-n. Crijaponine A, crijaponine B, ungeremine, lycorine, 2-*O*-acetyllycorine, 1,2-*O*-diacetyllycorine, (-)-crinine, 11-hydroxyvittatine, hamayne,(+)-epibuphanisine, crinamine, yemenine A, epinorgalanthamine. Criasiaticidine A, pratorimine, Lycorine, 4′-hyd’oxy-7-methoxyflavan. Crinamine, lycorine, norgalanthamine, epinorgalanthamine. Asiaticumines A, asiaticumines B.	Inhibitory activity against LPS-induced nitric oxide production. Anticancer activity (against cervical cancer SiHa cells). Inhibition of platelet aggregation. Promotion of hair growth through dermal papilla proliferation. Inhibition of the growth of HepG2 tumor cells. Anti-AChE activity, cytotoxic activity. Cytotoxic against Meth-A (mouse sarcoma) and Lewis lung carcinoma (mouse lung carcinoma). Inhibition of the activity of hypoxia inducible factor-1 (crinamine). Cytotoxicity.	[256,257,258,259,260,261,262,263,264,265,266]
*C. kirkii* Baker	Bulbs		Noraugustamine, 4a*N*-dedihydronoraugustamine, 3-*O*-acetylsanguinine, 1,2-diacetyllycorine.	Antiparasitic activity against *Trypanosoma brucei* (*T. brucei)* rhodesiense, *Trypanosoma cruzi* (*T. cruzi).*	[267,268]
*C. macowanii*	Bulbs		Macowine, lycorine, cherylline, crinine, krepowine, powelline, buphanidrine, crinamidine, undulatine, 1-epideacetylbowdensine, 4a-dehydroxycrinamabine.	NR.	[249]
*C. firmifolium*	Leaves	Madagascar	2-alkylquinolin-4(1H), 2-alkylquinolin-4(1H).	Antiplasmodial activity.	[269]
*C. latifolium*	Bulbs Leaves	China. Hanoi, Vietnam	4,8-dimethoxy-cripowellin C. 4,8-dimethoxy-cripowellin D, 9-methoxy-cripowellin B, 4-methoxy-8-hydroxy-cripowellin B, cripowellin C. *C.* latines A, *C.* latines B and *C.* latines C. 4-senecioyloxymethyl-3,4-dimethoxycoumarin, 5,6,3 ′-trihydroxy-7,8,4 ′-trimethoxyflavone. 4-methyloxysenecioyl-6,7-dimethoxycoumarin, 5,6,3′-trihydroxy-7,8,4′trimethoxyflavone.	Cytotoxic against tumor cell lines, antimicrobial activity, antioxidant activity. Inhibitory activity against human umbilical venous endothelial cells.	[270,271,272]
*C. scillifolium*	Bulbs		Scillitazettine, scilli-*N*-desmethylpretazettine.	Mild antiplasmodial activity	[273]
*C. zeylanicum* (L)	Bulbs, leaves, flowers, fruits	Cuba Sri Lanka	Crinine, Lycorine, 11-*O*-acetoxyambelline, ambelline, 6-hydroxybuphanidrine, 6-ethoxybuphanidrine, 3-acetylhamayne, 6-hydroxycrinamine, hamayne, 6-methoxycrinamine.	Antiproliferative effect.	[246,274]
*C. jagus* (J. Thomps) Dandy	Bulbs, leaves	Senegal Ghana	Gigantelline, gigantellinine, gigancrinine, sanguinine, cherylline, lycorine, crinine, flexinine, hippadine. Galanthamine, galanthamine *N*-oxide, powelline.	Anti-AChE activity, inhibitors of TcAchE, hAChE and hBChE	[275,276]
*C. abyscinicum* Hochst. ExA. Rich	Bulbs	Ethiopia	6-hydroxycrinamine, lycorine.	Antiproliferative activity against A2780 epithelial ovarian cancer and MV4-11 acute myeloid leukemia cell lines.	[277]
*C. erubescens*	Above ground plant parts	Puntarenas, Costa Rica	Cripowellin A, cripowellin B, cripowellin C, cripowellin D, hippadine.	Antiplasmodial activity.	[278]
*C. yemense*	Bulbs	Yemen	6-hydroxy-2H-pyran-3-carbaldehyde. Yemenines A, B and C, 1, (+)-bulbispermine, (+)-crinamine, (+)-6-hydroxycrinamine, (-)-lycorine.	Tyrosinase inhibitor. Inhibit nitric oxide production, induce nitric oxide synthase.	[277,278,279,280]
*C. bulbispermum* *C. bulbispermum* III	Bulbs	Egypt	8-hydroxylycorin-7-one, 2-deoxylycorine, vittatine, 11-hydroxyvittatine, hippamine. 4-hydroxy-2′,4′-dimethoxydihydrochalcone, 4,5-methylenedioxy-4′-hydroxy-2-aldehyde [1,1′-biphenyl], hippacine, 4′-hydroxy-7-methoxyflavan-3-ol, 2(S),3′,4′-dihydroxy-7-methoxy flavan, isolarrien, isoliquiritigenin, liquiritigenin. Bulbispermine.	NR.	[281,282,283]
*C. powellii*	Bulbs	Switzerland Colombia	Linoleic acid ethyl ester, alkaloid hippadine, calleryanin, 4′-hydroxy-7-methoxyflavan. Lycorine, 1-*O*-acetyllycorine, ismine.	AChE inhibitor (linoleic acid ethyl ester). Inhibition of topoisomerase 1 activity.	[284,285]
*C. glaucum*	Bulbs	Nigeria	Hamayne, lycorine, haemanthamane, crinamine.	Choline esterase inhibitory activity.	[286]
*C. purpurascens*	Leaves	Cameroon	4,5-ethano-9,10-methlenedioxy-7-phenanthridone, 4,5-ethano-9-hydroxy-10-methoxy-7-phenanthridone, α-d-glucopyranoside.	Antibacterial activity	[287]

NR: not reported.

**Table 12:** Geographical distribution of the Genus *Cyrtanthus*.

Lineage	Location	Species	References
Clade A	Southern Africa Grassland Southeastern African temperate grasslands Grassland of the Highveld in the northern parts Subtropical Indian Ocean Coastal Belt East Africa and Angola	*C. attenuatus*, *C. macowanii*, *C. epiphyticus*, *C. mackenii* subsp. *cooperi*, *C. huttonii*, *C. macmasteri*, *C. suaveolens*, *C. stenanthus* var*. stenanthus*, *C. flanaganii* *C. tuckii* var. *transvaalensis* *C. angustifolius*, *C. fergusoniae* *C. aureolinus*, *C. mackenii* subsp. M*ackenii*, *C. brachyscyphus* *C. breviflorus*	[328]
Clade B	Baviaanskloof Mountains and Eastern Cape (Fynbos and Albany Thicket Biomes) Semi-arid Succulent Karoo Greater Cape Region (“the Cape”) Coastal and inland mountains of the southern Cape Cape Peninsula into the Eastern Cape	*C. labiatus*, *C. montanus* *C. herrei* *C. carneus*, *C. elatus*, *C. guthrieae*, *C. labiatus*, *C. leptosiphon*, *C. leucanthus*, *C. montanus*, *C. odorus* *C. collinus* *C. ventricosus*	[328]
Clade C	Albany Thicket Biome Savanna Biome Northwards from the Albany region through South Africa, Zimbabwe, Western Mozambique and East Africa into Sudan Albany Thicket and Savanna Biomes Extends beyond the Savanna Biome into the Sub-Escarpment and Highveld grasslands Fynbos Biome Southern parts of the Nama Karoo	*C. flammosus*, *C. spiralis* *C. eucallus*, *C. galpinii* *C. sanguineus* *C. helictus* *C. contractus* *C. wellandii* *C. smithiae*	[328]

**Table 13:** A summary of the traditional uses, phytochemicals and pharmacological activities of *Cyrtanthus* species.

Plant Species	Traditional Uses	Compounds	Pharmacological Activities	References
*C. obliquus*	Chronic cough, headache and scrofula	5,7-dihydroxy-6-methoxy-3-(4′-methoxybenzyl)chroman-4-one, 5,7-dihydroxy-6-methoxy-3-(4′-hydroxybenzyl)chroman-4-one	Antioxidant activity	[338]
*C. contractus*	Mental illness, protective charm against evil spirits	NarciclasineNarciprimine	Anti-inflammatory activity (via inhibition of E-selectin, blockade of the expression of endothelial adhesion molecule ICAM-1)Acetylcholinesterase inhibitor	[337]
*C. breviflorus*	Emesis, worm infestations, protective charm against evil spirits	haemanthamine, lycorine, crinamine hydrochloride and tazettine	Antihelminthic	[337,342]
*C. elatus*	Cough, headache, labor induction	Haemanthamine, zephyranthine, galanthamine and 1,2-*O*-diacetylzephyranthine	Antiprotozoan activity, selective cytotoxic activity	[43,44,337]
*C. falcatus*	Not known to be used by the traditional South African people	Papyramine, epipapyramine, maritidine, *O*-methylmaritidine and tazettine	Antibacterial activity against *B. subtilis S. aureus* and *E. coli*, mutagenicity, cytotoxic activity	[342,343,344]
*C. suaveolens*	No traditional use has been reported	Captan	Mutagenicity, anti-inflammatory activity via inhibition of COX-2, fungicide	[342,344]

## Data Availability

Not applicable.

## Supplementary Materials

The following supporting information can be downloaded at: https://www.mdpi.com/article/10.3390/molecules27144475/s1, Table S1: Compounds from *Tulbaghia*. Reference [345] is cited in the Supplementary Materials.

## Abbreviations

MIC, minimum inhibitory concentration; MBC, minimum bactericidal concentration; MTT, 3-[4,5-dimethylthiazol-2-yl]-2,5 diphenyl tetrazolium bromide; MoA, mode of action; ROS, reactive oxygen species; TEAC, Trolox equivalent antioxidant capacity; FRAP, ferric-reducing antioxidant power; DPPH, 2,2-diphenyl-1-picryl-hydrazyl-hydrate; AAPH, 2,2′-azobis-2-amidinopropane dihydrochloride; TG, triglyceride; TC, total cholesterol; VLDL, very low-density lipoprotein; HDL, high-density lipoprotein; BP, blood pressure; AVD, atherothrombotic vascular disease; DCM, dichloromethane.
